# Decentralized fault-tolerant control of multi-mobile robot system addressing LiDAR sensor faults

**DOI:** 10.1038/s41598-024-75500-3

**Published:** 2024-10-28

**Authors:** Ahmed M. Elsayed, Mohamed Elshalakani, Sherif A. Hammad, Shady A. Maged

**Affiliations:** 1 Mechatronics Engineering Department, Higher Technological Institute, Tenth of Ramadan City, Al-Sharqia Egypt; 2 Mechatronics Engineering Department, Faculty of Engineering, Ain Shams University, Cairo, Egypt

**Keywords:** Dynamic threshold, DFTC, Graph theory, Lidar sensor fault, Sensor fusion, Engineering, Electrical and electronic engineering, Mechanical engineering

## Abstract

The control of multi-robot formations is a crucial aspect in various applications, such as transport, surveillance and monitoring environments. Maintaining robots in a specific formation pose or performing a cooperative task is a significant challenge when a fault occurs among any of the robots. This work presents a Decentralized Fault-Tolerant Control (DFTC) scheme that addresses lidar sensor faults within a system of multiple differential wheeled mobile robots. The robots change the formation shape according to the number of available robots within the formation. A Graph theory is implemented to represent the multi-robot formation and communication. Each mobile robot is equipped with three sensors: a wheel encoder, an Inertial Measurement Unit (IMU), and a lidar sensor. Sensor fault detection and isolation (FDI) is implemented at two levels. The pose estimation obtained from the wheel encoder and IMU is fused using an extended Kalman filter (EKF), and this pose estimation is utilized at the local level of lidar sensor FDI. At the system level, the FDI of the lidar sensor involves computing a residual by comparing the pose estimation with other lidar sensors mounted on other mobile robots within the formation. The presented FTC scheme is simulated in Simulink multi-robot environments.

The introduction of multi-robot systems has contributed to various sectors such as security, industrial 4.0 technologies, social, agricultural, and others. Multi-robot systems can perform more complex tasks efficiently than a single robot^1^. Some tasks require the robots to be in a specific geometrical formation. For instance, handling material with a particular shape and size in factories^2,3^. Mobile robot’s onboard sensors are significant in the perception of the surrounding environment, and their data is vital for formation control and coordination among the robots. Several sensors are used for localization and navigation depending on the locomotion of the mobile robot and whether the robots operate indoors or outdoors. For instance, GPS provides accurate measurements of the robot’s outdoor position but can not be used indoors. Also, IMU sensors are cheap and can be used in different environments, but they are subject to accumulating errors^4^. Many studies have investigated sensor fusion techniques for enhancing a robot’s state estimation^5,6^. Sensor fusion receives information from different sensors and combines them to eliminate distorted measurements and have more reliable information^4^. Fault occurrence is a significant challenge in multi-robot systems. A malfunction in one of the robots will subject the whole system to failure in the task. A fault occurrence could be in the actuator, sensor, or communication. Fault-tolerant control (FTC) is an approach that aims to create adjustable controllers that can maintain the performance of a closed-loop system at an acceptable level even when faults occur^7^.

## Related work

### Formation control

Different strategies and methodologies are applied to formation control. Each strategy has its merits and drawbacks^1^. presents a review of these strategies. It also highlights the challenges and problems of formation control, which can be classified as formation shape generation, formation shape tracking, formation shape reconfiguration, and task assignment information. A hybrid technique is used in which two formation control strategies are used. A study by^8^ used a Graph-based formation control to assess the formation stability, while the leader-follower strategy was used for formation trajectory tracking. Also, Analysis of the convergence of robots to a particular predefined shape is one of the formation control challenges. A work presented by^9^ proposed a decentralized formation scheme using Lyapunov techniques, considering local distance and orientation sensors. The control law used in this work includes integral-type terms that eliminate the error generated from the motor’s dead zone. A decentralized behaviour-based formation control algorithm for multiple robots considering obstacle avoidance is presented by^10^. This work achieves formation control using a behaviour-based algorithm based on the relative position between the robot and the neighbour. The robots do not keep formation until the obstacle avoidance task is completed. Obstacle avoidance behaviour is considered in this work using the concept of escape angle^11^. presents a formation control strategy on a group of mobile robots. The technique used is based on a consensus algorithm on a weighted graph. A weighted graph is used for collision avoidance within the robots. Formation control based on reinforcement learning was presented by^12^. The study considered homogeneous robots, and the model is unicycle. The learning process and execution both showed fluctuations, which were attributed to the non-stationarity of the environment concerning the agents. In contemporary discourse, there has been a discernible surge of interest in the comparative Analysis of formation control concerning model-based and learning-based methodologies^13^. present a study that introduces a formation control paradigm based on learning utilizing Deep Neural Networks (DNNs) and subsequently undertakes a comparative analysis for a model-based approach. The study shows that the model-based approach is applicable when the dynamic / kinematics are known and the environment dynamics are simple. However, learning-based can deal with complex dynamics on the condition that the training data are available. The author^14^ presented an overview of advancement in LiDAR-based global localization, including cross-robot localization methods. Different techniques of loop closure detection in multi-robot systems. Furthermore, author^15^ introduced a LiDAR-based localization method for multi-robot formation control. The algorithm utilized in this study employs DBSCAN clustering to identify potential robots. Notably, the proposed method operates without the need for inter-robot communication. However, it exhibits certain limitations, such as reduced accuracy when robots are positioned at specific angles relative to each other. A distributed collaborative LiDAR SLAM framework is present by^16^, this method used to co-localize in an unknown environment with low information exchange within a robotic swarm. The proposed DCL SLAM method adopted LiDAR-Iris^17^ descriptor for efficient place recognition. This work achieved better accuracy than single robot SLAM and aims to test the system with a solid-state LiDAR.

### Sensor fusion

Sensor fusion techniques are used to enhance both navigation and perception of the surrounding complex environment, in addition to overcoming the drawbacks of other sensors. A study by^6^ presents a mobile robot indoor position system using IMU, UWB (Ultra Wide Band), and odometry. This work implements a sensor fusion between IMU and WMB based on the EKF algorithm. Then, fuse the output with odometry using complementary filtering to increase efficiency. The result shows less RMSE using the Fusion algorithm. Moreover^18^, proposed a navigation system that fused inertial measurement unit (IMU) data, camera imagery, and wheel encoder measurements. This research utilized an EKF algorithm to integrate the pose estimates from the three sensors mentioned above to localize the robot. Sensor fusion is classified based on the input provided to the fusion framework, wherein low-level fusion concerns the integration of raw sensor data, medium-level fusion involves the fusion of extracted features derived from raw data, and high-level fusion addresses decision fusion^19,20^. A study by^21^ used a position algorithm based on IMU/Odometer/Lidar; in this work, a Kalman filtering algorithm was used to fuse an odometer-assisted system and lidar feature extraction to obtain a real-time robot position. The author^22^ introduced a positioning methodology for indoor mobile robots, employing a multi-sensor fusion factor graph (MSF_FG). This investigation incorporated an inertial measurement unit (IMU), odometry, and light detection and range (LiDAR) sensors. The findings revealed a 40% reduction in mean location error compared to conventional inertial navigation systems and the extended Kalman filter algorithm.

### Fault tolerant control

Identifying and diagnosing faults constitute a pivotal stage within fault-tolerant control systems. Fault detection could be for actuators or sensors. A study by^23^ proposed an interacting multiple-model (IMM) approach to sensor fault and identification (FDI) in the dead reckoning of mobile robots. This work used probability averaging and heuristic design, making the sensor normal/failure mode switching rules. In addition, the work considers only the hard sensor failures. A work by^24^ proposed an informational approach for sensor and actuator fault diagnosis for autonomous mobile robots. A Kullback-Leibler divergence is used for residual computing. A work addresses fault-tolerant cooperative control of multiple wheeled mobile robots proposed by^25^. This work presents actuator fault detection and diagnosis using two two-stage Kalman filters. The leader-follower technique is used for shape formation. The author^26^ proposed a survey on Fault detection, isolation, and identification for lidar. This work presented a classification of perception sensor fault and a classification of FDII methods for perception sensors. In addition, a work by^27^ proposed a real-time fault-tolerant formation control for multiple WMRs. The implemented strategy is concerned with severe actuator faults. A hybrid genetic algorithm and particle swarm optimization technique were used to reconfigure the trajectory of the remaining healthy robots. Mobile robots are subjected to the risk of cyber/physical attacks and software/hardware failures. Another study proposed by^28^ introduced an actuator fault-tolerant control scheme for differential-drive mobile robots based on a multiple-model approach. In this research, a bank of EKF is designed for actuator fault detection and isolation; the proposed approach is validated experimentally using QBot2 robot. A distributed leader-following fault-tolerant tracking control is proposed by^29^. A learning approach based on NN is used to learn unknown faults and ensure system stability^30^. Proposed a technique to identify anomalies in sensors and actuators by utilizing the physical dynamics of mobile robots using a model-based estimation approach. This research focuses on detecting misbehaviour rather than identifying its origins. Furthermore, different research investigated the topic of fault-tolerant formation control concerning actuator faults^31–35^. Concerning the topic of FTC in multi-robot systems, a work by^36^ presented a sensor fault detection in a multi-robot team, the approach of fault detection in this work used hardware redundancy by comparing velocity and position estimates from multiple sources (wheel encoder, gyro and laser scan). Furthermore, a work presented by^37^ proposed a technique based on a distributed controller-observer architecture that allows each agent to estimate the global system using local communication. Work by^38^ presents a fault-tolerant safe control framework for a LiDAR-based system that addresses proprioceptive and exteroceptive sensor faults and attacks, also the accuracy of the LiDAR scan is validated by comparing them with the reconstructed version. The authors in this work validate the proposed framework using UAV.

### Contribution

The aforementioned literature reveals a research gap in the domain of fault-tolerant control in multi-robot system concerning Lidar sensor faults. This paper introduces a decentralized fault-tolerant control methodology for a multi-mobile robot system experiencing Lidar sensor malfunctions. The proposed method employs a leader-follower formation control strategy. To ensure formation stability, a graph-theoretic approach is incorporated. The robot’s obstacle avoidance behaviour is regulated using a vector-field histogram technique^39^. Various methodologies exist for applying Fault Detection and Isolation (FDI) to LiDAR sensors. One such technique involves the computation of sensor anomalies, which can be implemented by comparing LiDAR data to a specific sensor model, or another sensor of the same or different type. Additional methods include comparisons to a state of static ground truth or dynamic truth. A comprehensive survey of these FDI methodologies as applied to LiDAR sensors is presented in the referenced work^26^. The current work presents an approach that implements two FDI methods for the lidar sensor of each robot. The first method operates at the local level, computing residuals by comparing the lidar sensor data with data from different sensor modalities onboard the robot. Specifically, an Inertial Measurement Unit (IMU) and wheel encoders are utilized for robot pose estimation. The data from the IMU and wheel encoders are fused using an extended Kalman filter (EKF). The second method is implemented at the multi-robot system level, involving the computation of residuals by comparing the lidar sensor data with lidar sensors of the same type mounted on other robots within the formation. When one of the robots encounters a sensor error, it isolated from the formation and so the number of available robots in the formation is modified. In addition, a formation reconfiguration is done and the Hungarian algorithm is applied for task assignment cost reduction. This approach provides a comprehensive framework for DFTC in multi-robot systems concerning Lidar sensor faults. The communication technique and data transfer within the multi-robot system is not present since it is not the scope of this work.

## Paper organization

The remainder of this paper is organized into the following sections. The models and methods section details the robot model and sensor measurement models utilized in this work. Multi-robot group formation section illustrates a strategy for multi-robot formation control. Subsequently, the decentralised fault-tolerant control section examines the DFTC approach and fault detection and isolation (FDI) methodology. Finally, the Simulation section illustrates the simulation and results, a Matlab/Simulink multi-robot environment is used for implementation of the control strategy presented in this work.

## Models and methods

In this study, three distinct models are introduced. The first is a mathematical model specifically designed for a differential wheel mobile robot. Additionally, we present models for the onboard sensors, which include the wheel encoder, the Inertial Measurement Unit (IMU), and the Light Detection and Ranging (Lidar) system. These models serve as the foundation for our subsequent analysis and discussion.

### Wheeled mobil robot kinematic

**Fig. 1:**
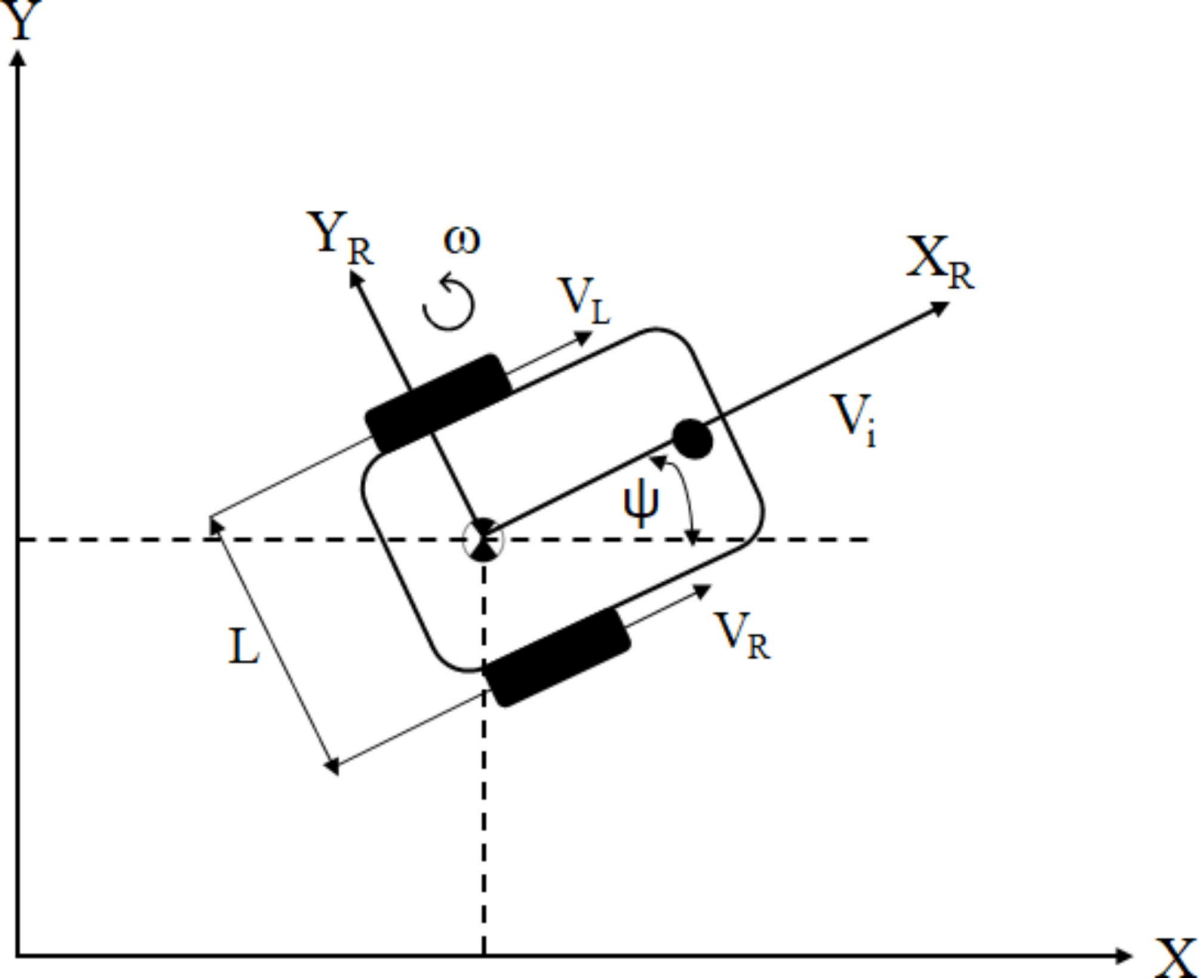
Wheeled mobile robot kinematics.

The research focuses on a multi-robot system comprising five mobile robots equipped with differential wheel drive systems. The motion of the differential wheel drive mobile robot in a two-dimensional space is depicted in Fig. 1. The kinematics model is shown in Eq. (1) as written in^40^. All the robots in the system have the same model since they are identical.

Where V_R_ and V_L_ are the velocities of the right and left wheels respectively, v is the robot’s linear velocity and ω is the robot’s angular velocity, which can be the control inputs. L is the robot’s wheelbase, $x$ and $y$ are the robot’s position, $\theta$ is the robot orientation. Both robot’s position and orientation represent the robot state in the global frame. Thus, the robot coordinate vector and control input vector can be described as: q(t) = [x(t), y(t), θ(t)]^T^ and u(t) = [v(t), ω(t)]^T^.

1 $$v=\frac{{\text{V}}_{\text{R}}+{\text{V}}_{\text{L}}}{2},{\upomega}=\frac{{\text{V}}_{\text{R}}+{\text{V}}_{L}}{\text{L}},\dot{q}=\left[\begin{array}{cc}\text{cos}\left({\uppsi}\right)&0\\ \text{sin}{\uppsi}&0\\ 0&1\end{array}\right]\left[\begin{array}{c}v\\ {\upomega}\end{array}\right]$$

The angular velocity of each wheel can be calculated as follows. Where r is the wheel’s radius

2 $${{\upomega}}_{\text{R}}=\frac{{\text{V}}_{\text{R}}}{\text{r}},{{\upomega}}_{\text{L}}=\frac{{\text{V}}_{\text{L}}}{\text{r}}$$

### Sensor measurement model

The multi-robot system in the current study utilizes a mobile robot equipped with an Inertia Measurement Unit (IMU) and wheel encoder. The wheel encoder and IMU sensors will be used subsequently in FDI of the lidar sensor defects.

#### IMU sensor

IMU sensor includes a 3-axis accelerometer and 3-axis gyroscope. The accelerometer measures the robot acceleration in x(a_x_), y(a_y_), and z(a_z_) axis, assuming a_z_ to be zero since the robot movement is in 2D space. Accelerometer data can be integrated to calculate the robot’s location. However, the gyroscope measures the robot’s angular velocity in three axes ($\dot{{\upphi}}$, $\dot{{\uptheta}}$, $\dot{{\uppsi}}$), and can be integrated to calculate robot orientation. The IMU equation can be written as in^4^:

3 $$\left[\begin{array}{c}\dot{X}\\ \dot{Y}\\ \dot{Z}\\ \dot{U}\\ \dot{V}\\ \dot{W}\\ \dot{{\upphi}}\\ \dot{{\uptheta}}\\ \dot{{\uppsi}}\end{array}\right]=\left[\begin{array}{c}\left[\begin{array}{c}U\\ V\\ W\end{array}\right]\\ {R}_{3\times3}\left\{\begin{array}{c}{a}_{x}\\ {a}_{y}\\ {a}_{z}\end{array}\right\}\\ \left[\begin{array}{ccc}1&\text{sin}{\upphi}\text{tan}{\uptheta}&\text{cos}{\upphi}\text{tan}{\uptheta}\\ 0&\text{cos}{\upphi}&-\text{sin}{\upphi}\\ 0&\text{sin}{\upphi}\text{sec}{\uptheta}&\text{cos}{\upphi}\text{sec}{\uptheta}\end{array}\right]\left[\begin{array}{c}p\\ q\\ r\end{array}\right]\end{array}\right]$$

where U, V, and W are the robot linear velocities in the x, y and z axis respectively. However, p, q, and r are robot angular velocities in the x, y, and z axis respectively.

#### Wheel encoder

The present research employs a differential wheel-drive mobile robot (DWDM), in which the wheels operate independently of each other. Every wheel is fitted with a wheel encoder that has a resolution of 1000 pulses per revolution. The wheelbase is assumed to be 18 cm. The wheel encoder measurement model may be expressed based on the robot kinematic model.

4 $$\left[\begin{array}{c}\dot{X}\\ \dot{Y}\\ \dot{{\uppsi}}\end{array}\right]=\left[\begin{array}{c}\frac{{V}_{R}+{V}_{L}}{2}\text{cos}{\uppsi}\\ \frac{{V}_{R}+{V}_{L}}{2}\text{sin}{\uppsi}\\ \frac{{V}_{R}-{V}_{L}}{L}\end{array}\right],{\text{V}}_{\text{R}}=\frac{2{\uppi}{\varDelta\text{ticks}}_{\text{R}}}{\text{resolution}*\text{dt}},{\text{V}}_{\text{L}}=\frac{2{\uppi}{\varDelta\text{ticks}}_{\text{L}}}{\text{resolution}\text*\text{dt}}$$

where Ẋ and Ẏ are the rate of change of robot position in x and y coordinates respectively. However, the $\dot{{\uppsi}}$ is the rate of change of robot orientation.

#### Lidar

Lidar sensors measure distance by illuminating a target with a laser and analysing the time of reflected light. The lidar model belongs to three categories: beam-based, scan-based and landmark-based^41^. Multi-beam rotating LiDAR sensors are popular for use in autonomous mobile mapping and robotics. It can generate a 2D or 3D map of cloud points representing the surrounding environment. Each laser point has an azimuth angle $\left(\alpha\right)$, elevation angle ($\epsilon)$ and range (R), those values are used to compute and coordinate the laser point. In the case of a 2D lidar, the elevation angle is considered to be zero. The point coordinates can be calculated using the following equation as stated in^42^:

5 $${point}_{x}=R*\text{cos}\left(\epsilon\right)*\text{sin}\left(\alpha\right){point}_{y}=R*\text{cos}\left(\epsilon\right)*\text{cos}\left(\alpha\right){point}_{z}=R*\text{sin}\left(\epsilon\right)$$

## Multi-robot group formation

This work uses a multi-robot system consisting of five DWM robots. Implementing a decentralized control strategy to coordinate the robot’s arrangement into a geometric shape formation. The applied technique in this study for shape development is the leader-follower approach combined with graph theory. The leader-follower system is used for route-following in formations. Graph theory is used to establish the desired relative distance and orientation between the leader robot and the other following robots. Furthermore, graph theory is used to assess the stability of formations.

**Fig. 2:**
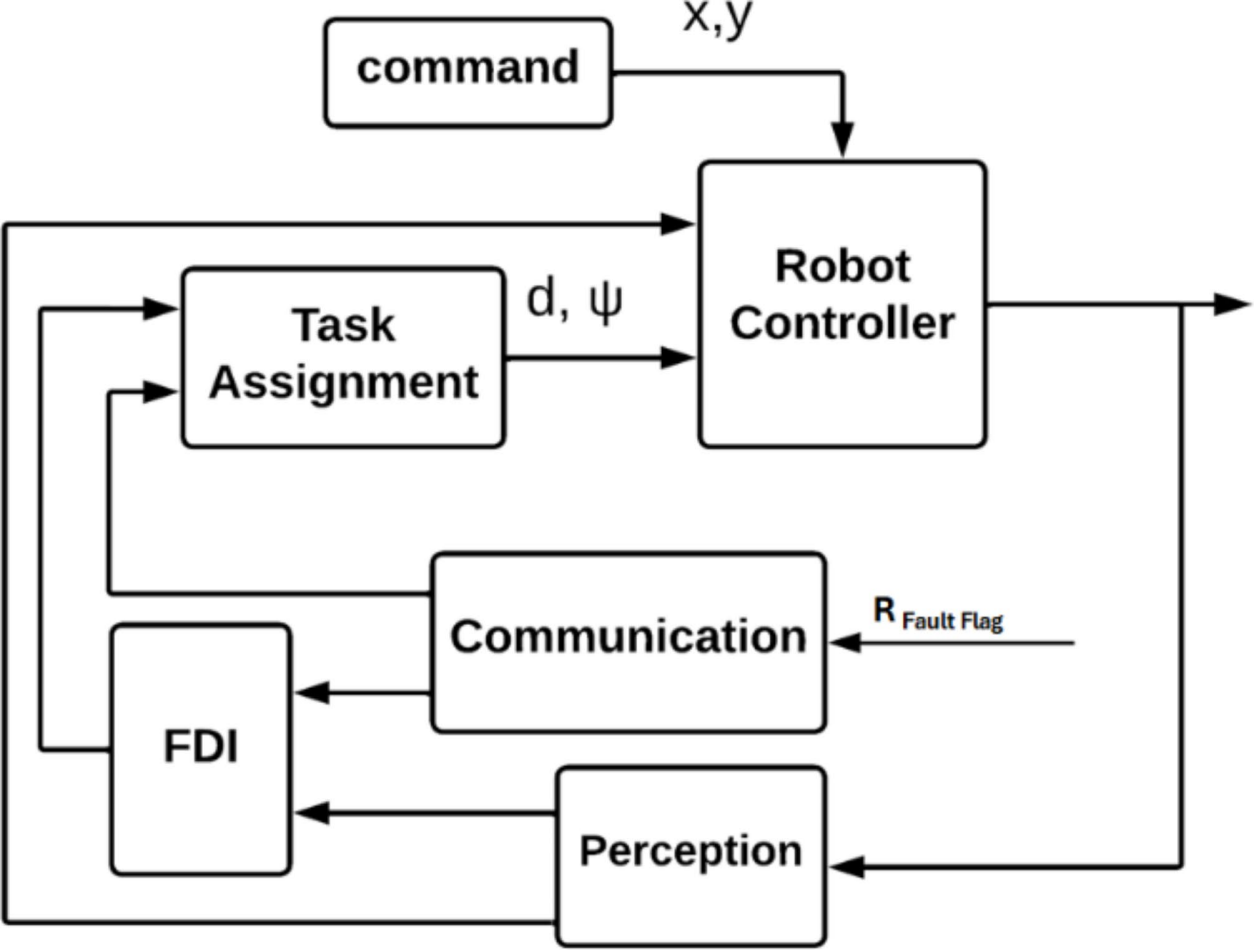
Mobile robot control system block diagram.

Figure 2 shows a block diagram of the control system for a single agent. The perception subsystem of the mobile robot incorporates a comprehensive state localization module. The localization process employs a multi-sensor fusion approach, integrating data from the Inertial Measurement Unit (IMU) and wheel encoders through an Extended Kalman Filter (EKF) framework. This sensor fusion is further augmented by incorporating LIDAR-based localization techniques. However, the Fault Detection and Isolation (FDI) module is responsible for detecting and isolating faults or anomalies in the robot’s LiDAR sensors. Upon identifying a LiDAR sensor fault, the FDI algorithm isolates the affected robot from the formation to prevent propagation of erroneous data and determines the number of operational robots remaining within the formation. FDI algorithm discussed in detail in the FTC section The communication block in the diagram represents the interaction between a single agent and the other agents in the formation. The function of communication is described in section five.

### Graph theory

**Fig. 3:**
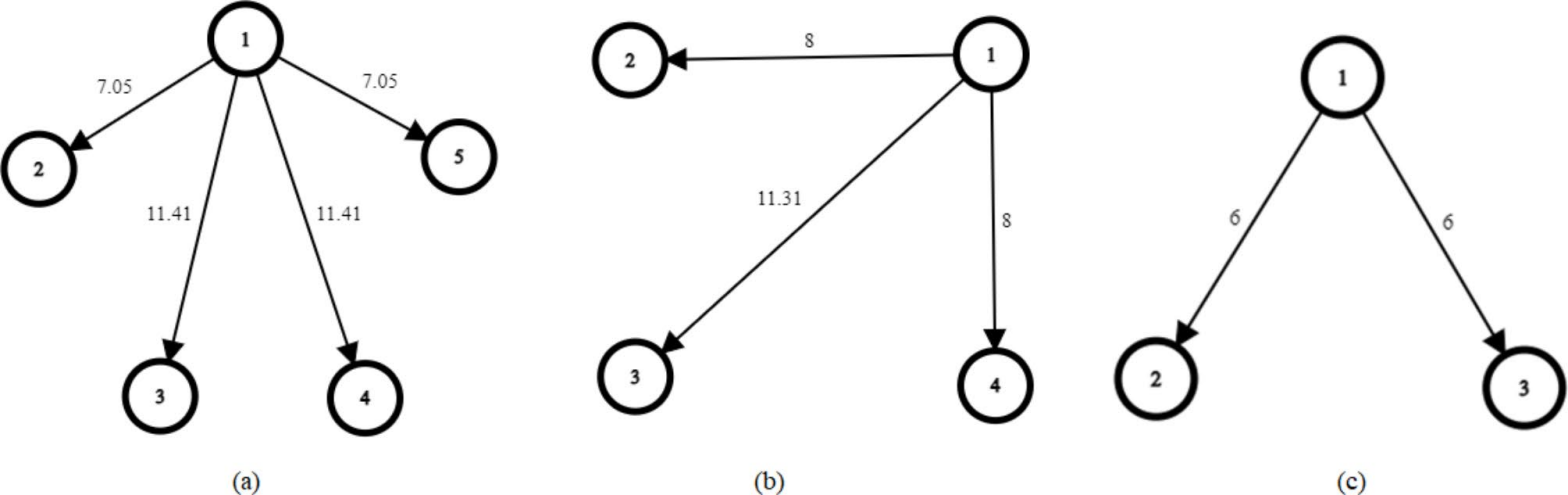
(**a**) Pentagon formation graph, (**b**) Square formation graph, (**c**) Triangle formation graph.

The undirected graph $$\mathcal{G}$$ is a pair of ($$\mathcal{V}$$,$$\mathcal{}\mathcal{E}$$).$$\mathcal{V}$$ is the set of vertices; $$\mathcal{V}=\left[{\upsilon}_{1},{\upsilon}_{2},\dots,{\upsilon}_{n}\right]$$and (n) is the number of nodes. And $$\mathcal{E}$$ is the set of undirected edges where $$\mathcal{E}\subseteq\mathcal{}\mathcal{V}\times \mathcal{V}$$. The edges connect a pair or vertex, such that if the vertex pair ($$i$$, $$j$$) $$\in$$$$\mathcal{E}$$ then so is ($$j$$, $$i$$). The number of edges $$l$$, where $$l\in\left\{1,\dots,\frac{n\left(n-1\right)}{2}\right\}$$. The set of neighbours of vertex $$i$$ is represented by $${\mathcal{N}}_{i}\left(\mathcal{E}\right)=\left\{j\in\mathcal{V}\mathcal{}|\mathcal{}\left(i,j\right)\in\mathcal{}\mathcal{E}\right\}$$. The *adjacency matrix*$$\mathcal{A}=\mathcal{}\left[{a}_{ij}\right]\in{\mathbb{R}}^{n\times n}$$ regarding a graph $$\mathcal{G}=\left(\mathcal{V},\mathcal{}\mathcal{E}\right)$$ is defined as $${a}_{ij}=1$$ if $$\left(i,j\right)\in\mathcal{E}$$, otherwise $${a}_{ij}=0$$. Also $${a}_{ij}={a}_{ji}$$, $$i\ne j$$ and $${a}_{ii}=0$$. And the Laplacian matrix $$\mathcal{L}=\mathcal{}\left[{\mathcal{l}}_{ij}\right]\in{\mathbb{R}}^{n\times n}$$ is defined as $${\mathcal{l}}_{ii}=\sum_{j=1`}^{n}{a}_{ij}$$ and $${\mathcal{l}}_{ij}=-{a}_{ij},i\ne j$$. More detail regarding graph rigidity theory can be found in^43^. Within the scope of this research, the theory of graph rigidity is employed as a framework for the representation of multi-robot formation and communication. The vertices of the graph, or nodes, are symbolic representations of mobile robots. The edges of the graph, however, vary depending on whether the graph is a formation or communication graph. Given that the formation strategy adopted in this study is a leader-follower strategy, the edges in the formation graph denote the intended distance between the leader and the follower. Furthermore, these edges are allocated weights that correspond to the intended distance. The geometric formations designated in this study include pentagon, square, and triangular shape configurations, as shown in Fig. 3 (a), (b) and (c) respectively. The decision to choose the shape formation configuration depends on the number of available robots in the system.

The adjacency matrix of the formation shape configurations directed graph is as follows:

6 $${\mathcal{A}}_{Pentagone}=\left[\begin{array}{ccccc}0&0&0&0&0\\ 1&0&0&0&0\\ 1&0&0&0&0\\ 1&0&0&0&0\\ 1&0&0&0&0\end{array}\right],\quad {\mathcal{A}}_{Square}=\left[\begin{array}{cccc}0&0&0&0\\ 1&0&0&0\\ 1&0&0&0\\ 1&0&0&0\end{array}\right], \quad {\mathcal{A}}_{Trianlge}=\left[\begin{array}{ccc}0&0&0\\ 1&0&0\\ 1&0&0\end{array}\right]$$

**Fig. 4:**
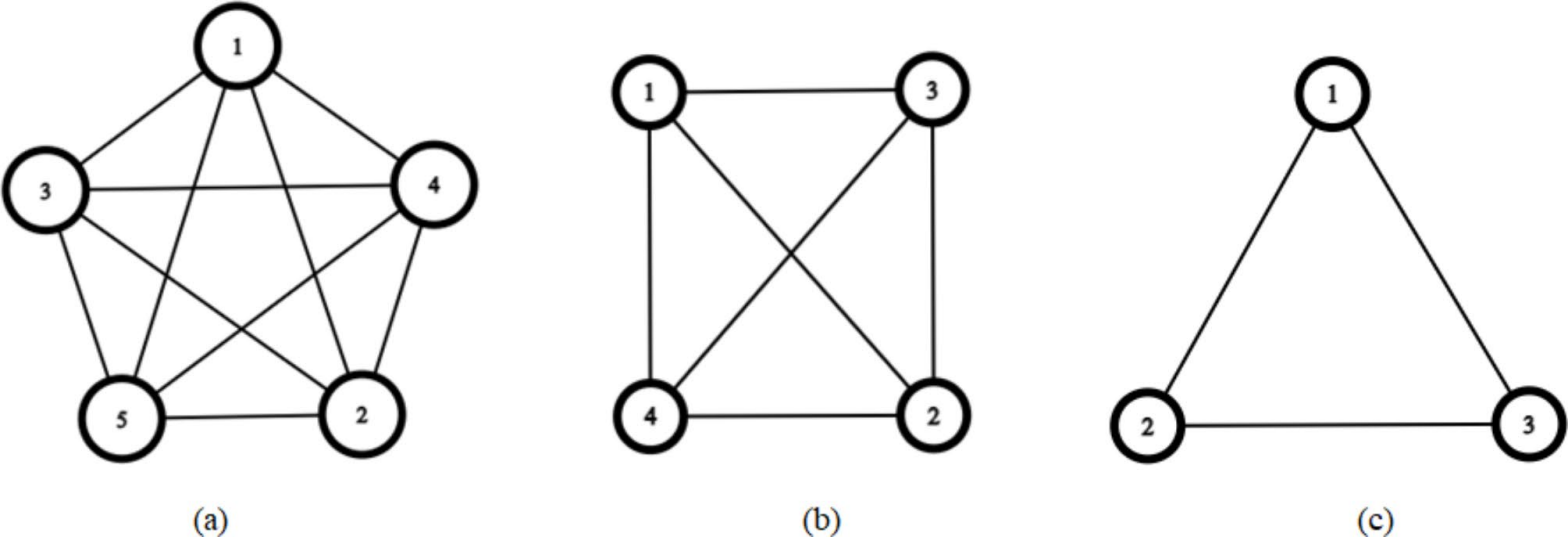
(**a**) Pentagon communication graph, (**b**) Square communication graph, (**c**) Triangle communication graph.

For designing a decentralized fault-tolerant control approach, each robot needs information about other robots in the formation allowing distributed design making. The communication structure among robots is represented by another communication undirected graph, where nodes are robots and edges denote communication links. This graph is essential for information exchange among robots. The undirected graph representing the communication among the robots is shown in Fig. 4.

The laplacian and adjacency matrices of the undirected graph representing the communication in the case of pentagon, square and triangle formation are presented in equations, ( 7), ( 8), and ( 9) respectively.

7 $${\mathcal{A}}_{Pentagone}=\left[\begin{array}{ccccc}0&1&1&1&1\\ 1&0&1&1&1\\ 1&1&0&1&1\\ 1&1&1&0&1\\ 1&1&1&1&0\end{array}\right]{\mathcal{L}}_{Pentagone}=\left[\begin{array}{ccccc}4&-1&-1&-1&-1\\ -1&4&-1&-1&-1\\ -1&-1&4&-1&-1\\ -1&-1&-1&4&-1\\ -1&-1&-1&-1&4\end{array}\right]$$

8 $${\mathcal{A}}_{Square}=\left[\begin{array}{cccc}0&1&1&1\\ 1&0&1&1\\ 1&1&0&1\\ 1&1&1&0\end{array}\right]{\mathcal{L}}_{Square}=\left[\begin{array}{cccc}3&-1&-1&-1\\ -1&3&-1&-1\\ -1&-1&3&-1\\ -1&-1&-1&3\end{array}\right]$$

9 $${\mathcal{A}}_{Triangle}=\left[\begin{array}{ccc}0&1&1\\ 1&0&1\\ 1&1&0\end{array}\right]{\mathcal{L}}_{Triangle}=\left[\begin{array}{ccc}2&-1&-1\\ -1&2&-1\\ -1&-1&2\end{array}\right]$$

### Task assignment

In the present study, a decentralized Fault-Tolerant Formation Control (FTFC) algorithm is employed, thereby enabling each robot to possess its Fault Detection and Isolation (FDI) and task assignment algorithm. Also, assuming there is no fault in communication between the robots. In the previous section, shape formation configuration is determined based on the number of available robots as follows.

$$n=\left\{\begin{array}{cc}5&,pentagonconfig\\ 4&,squareconfig\\ 3&,triangleconfig\end{array}\right.$$

A vector of distances, denoted as D, is constructed and includes the linear distances between the current location of the robot and the other target positions within the formation. Since a leader-follower formation control strategy is employed the D size is n-1. A Hungarian algorithm is used to optimally assign robots to positions in the formation. Let’s denote the cost matrix for the assignment problem as C, where C_ij_​ represents the cost of assigning follower *i* to position *j*. The cost matrix size is ${n}_{p}\times {n}_{p}$, where ${n}_{p}$ is the number of positions derived from the D. Hence, the dimensions of the cost matrix vary with each distinct shape formation configuration. The cost matrix designed for the assignment of positions to achieve a pentagonal formation is presented as follows:

10 $$C=\left[\begin{array}{cccc}{c}_{11}&{c}_{12}&{c}_{13}&{c}_{14}\\ {c}_{21}&{c}_{22}&{c}_{23}&{c}_{24}\\ {c}_{31}&{c}_{32}&{c}_{33}&{c}_{34}\\ {c}_{41}&{c}_{42}&{c}_{43}&{c}_{44}\end{array}\right]$$

## Decentralised fault-tolerant control

**Fig. 5:**
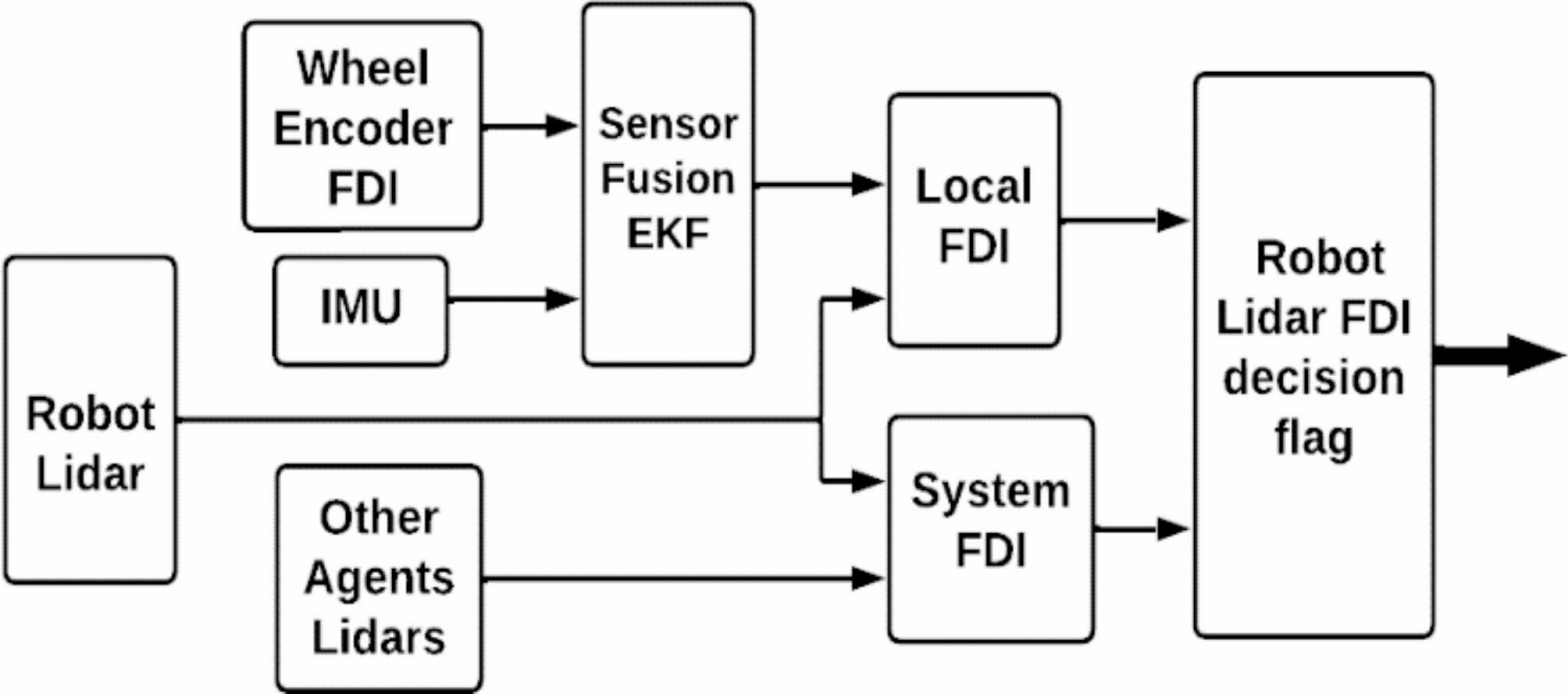
Lidar FDI system.

In the present work fault tolerant control is employed to a group of autonomous agents cooperating to achieve a common goal. A differential drive-wheeled mobile robot is utilized in this work. The term Fault tolerant concerns the ability of the multi-robot system to continue operation in the presence of faults. The fault could be an actuator, sensor or communication fault. The scope of this research is sensor fault in lidar. Fault detection and isolation (FDI) is implemented through two distinct stages: the local stage and the system stage. The local stage focuses on computing residuals between the Lidar sensor and other onboard sensor types within the robot. However, the system stage involves computing residuals using the same sensor type mounted on other robots in the system. Figure 5 depicts the Lidar FDI proposed in the current study.

### IMU/wheel encoder fusion

Sensor fusion is employed to integrate data from an Inertial Measurement Unit (IMU) and wheel encoder, thereby enhancing pose estimation accuracy. This fusion methodology addresses issues such as IMU drift reduction and the correction of wheel slippage or skidding. The fusion of IMU and wheel encoder data is implemented using Extended Kalman Filter (EKF). Within the EKF framework, IMU measurements, encompassing accelerations and angular velocities are assimilated to correct orientation and heading. Concurrently, wheel encoder measurements, involving the conversion of wheel rotations into distance changes, contribute to updating the position estimate. In EKF the system state transition probability and the measurement probability are described by a nonlinear function $g$ and $h$ respectively:

11 $${x}_{t}=g\left({u}_{t-1},{x}_{t-1}\right)+{\epsilon}_{t},{z}_{t}=h\left({x}_{t}\right)+{\delta}_{t}$$

### Fault detection and isolation

Various fault detection and isolation (FDI) methods for LiDAR sensors have been proposed in the literature^26^. Two of these methods are implemented in the present work. The first method involves comparing the LiDAR sensor measurements with measurements obtained from a different type of sensor. In this case, the alternative measurement is a fused estimate derived from wheel encoders and an inertial measurement unit (IMU). This method is implemented at the local level, where all sensors are on board the same mobile robot. On the other hand, the second method entails comparing the LiDAR measurements with measurements from another LiDAR sensor of the same type. This method is implemented at the system level, as the comparison is performed between LiDAR sensors mounted on multiple mobile robots operating in a formation. At the local level, the implementation of Lidar sensor fault detection is carried out in a series of sequential steps.

- The initial step is the fault detection process to the wheel encoder, the residual is computed between the robot pose estimation from the wheel encoder pulses and the state estimation from the EKF, assuming the robot moving in a 2D space the transition function can be defined as follows:
  12 $${x}_{t+1}={x}_{t}+{v}_{t}.\varDelta t.\text{cos}\left({\theta }_{t}\right){y}_{t+1}={y}_{t}+{v}_{t}.\varDelta t.\text{sin}\left({\theta }_{t}\right){\theta }_{t+1}={\theta }_{t}+{\omega}_{t}.\varDelta t$$
- If the wheel encoder is devoid of faults, a sensor fusion process is implemented between the wheel encoder and the Inertial Measurement Unit (IMU). This fusion process enhances the estimation of the robot’s pose by mitigating the drift error inherent in the IMU and the error caused by the skidding or slipping of the robot’s wheels.
  The system state uncertainty of the robot is represented by the covariance matrix from the extended Kalman filter as shown in Eq. (13). The covariance matrix captures the uncertainties associated with the robot’s state variables, including its position and orientation. Where ${\sigma}_{x}^{2}and{\sigma}_{y}^{2}$ represent the variances of the robot’s position in x and y respectively, and ${\sigma}_{\theta }^{2}$ represent the variance of robot’s orientation angle. Those values indicate the uncertainty of the robot’s position and orientation.
  13 $$P=\left[\begin{array}{ccc}{\sigma}_{x}^{2}&{\sigma}_{xy}&{\sigma}_{x\theta }\\ {\sigma}_{yx}&{\sigma}_{y}^{2}&{\sigma}_{y\theta }\\ {\sigma}_{\theta x}&{\sigma}_{\theta y}&{\sigma}_{\theta }^{2}\end{array}\right]=\left[\begin{array}{ccc}0.04153&0&0\\ 0&0.04153&0\\ 0&0&0.06136\end{array}\right]$$
- The final stage involves the computation of the residual between the fused data from the IMU and encoder, and the pose estimation derived from the Lidar measurement. If the residual surpasses a certain threshold, a Lidar fault flag is generated.
  14 $${r}_{l}\left(t\right)=\left|{L}_{pose}\left(t\right)-{F}_{pose}\left(t\right)\right|$$
  Fault detection and isolation (FDI) at the system level involves detecting anomalies in lidar sensors by computing residuals of pose estimation from onboard lidar and lidars mounted on other available robots in the formation, followed by averaging these residuals (15). Similar to the local level, if the residual exceeds the defined threshold, a fault flag is triggered.
  15 $${{r}_{s}\left(t\right)}_{avg}=\frac{1}{n-1}\sum_{i=1}^{n-1}{p}_{1}-{p}_{i}$$
  The threshold used in this study is dynamic and is determined based on the probability of cluttering in the surrounding environment. The dynamic threshold can be defined as follows:
  16 $${\uptau}=\text{ACC}\times \left(1+\text{K}\times {p}_{occu}(\text{m}\left(\text{t}\right),{p}_{r}(\text{t})\right)$$
  where; ACC is the lidar accuracy, and it defines the threshold baseline. The sensor accuracy is set by the manufacturer. K is a scaling factor that determines the density of clutter. A high clutter will result in a larger threshold for sparser environments and it is defined as in Eq. (17). ${p}_{occu}$ is a function that return the probability occupancy value in the occupancy grid map (m) at the robot pose ${p}_{r}$ at time (t). The function return values between 0 (free) and 0.9 (occupied).
  17 $$k=\frac{{\tau}_{{max}}-{\tau}_{{min}}}{1-{P}_{occu}}$$
  If a fault flag associated with a lidar is activated at both local and system levels, it serves as an indication of a defect within the lidar. To ascertain the existence of a sensor fault within the system, and to eliminate any prevailing uncertainty a residual moving average ${(r}_{MA\left(t\right)})$ technique is employed, if ${r}_{MA\left(t\right)}$ is greater than the threshold a fault flag is triggered as described in Eq. (18). Consequently, the robot identified as faulty is removed from the formation, prompting the remaining operational robot to reconfigure the formation.
  18 $${r}_{MA\left(t\right)}=\frac{1}{n}{\sum}_{i=0}^{n-1}{P}_{t-i}{fault}_{flag}=\left({r}_{MA\left(t\right)}>\tau\left(t\right)\right)?1:0$$

### Lidar faults simulation

Lidar sensors are susceptible to various types of faults as mentioned in^26^. In this study, the faults simulated include (1) environmental conditions, (2) mounting issues, and (3) defects in sensor subcomponent.

**Fig. 6:**
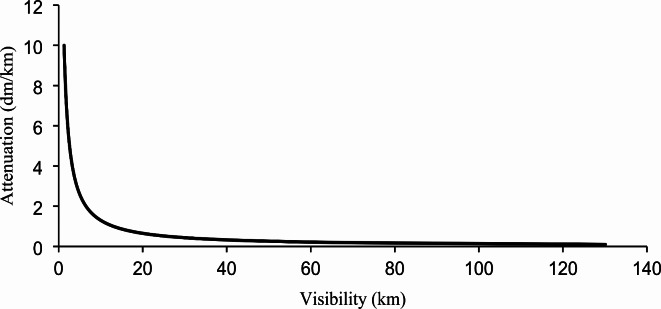
Smoke attenuation vs. visibility.

Environmental conditions can induce various faults in robotic systems. In this study, we focus on simulating the presence of smoke in the air, which introduces noise into LIDAR measurements. This noise can manifest through two primary mechanisms: (1) scattering of the laser beam by smoke particles, and (2) attenuation of the LIDAR signal. Our work specifically simulates the attenuation of the LIDAR signal caused by reduced visibility due to airborne smoke particles. The effect of smoke on visibility can be quantified using Eqs. (19) and (20) as given in^44^. where V represents visibility (in km), T is the transmittance, L is the propagation distance (in km), and β is the attenuation coefficient (in dm/km) [37]. The attenuation coefficient β is calculated using the Beer-Lambert law^45^, which relates the transmittance (T) and propagation distance (L). The attenuation process can be modelled as an exponential decay function, where the intensity of the returned signal decreases with increasing smoke density^46^. Figure 6 shows the attenuation coefficient and visibility simulated in this study. In this study, the assumed measurement noise distribution resulting from variations in attenuation is modelled as a random distribution.

19 $$v=-\frac{10{log}_{10}\left({T}_{th}\right)}{{\beta}_{\lambda}}$$

20 $${\beta}_{\lambda}=-\frac{10{log}_{10}\left(T\right)}{4.343L}$$

21 $$T\left(L\right)=\frac{I\left(L\right)}{I\left(0\right)}=\text{exp}(-{\alpha}_{e}L)$$

**Fig. 7:**
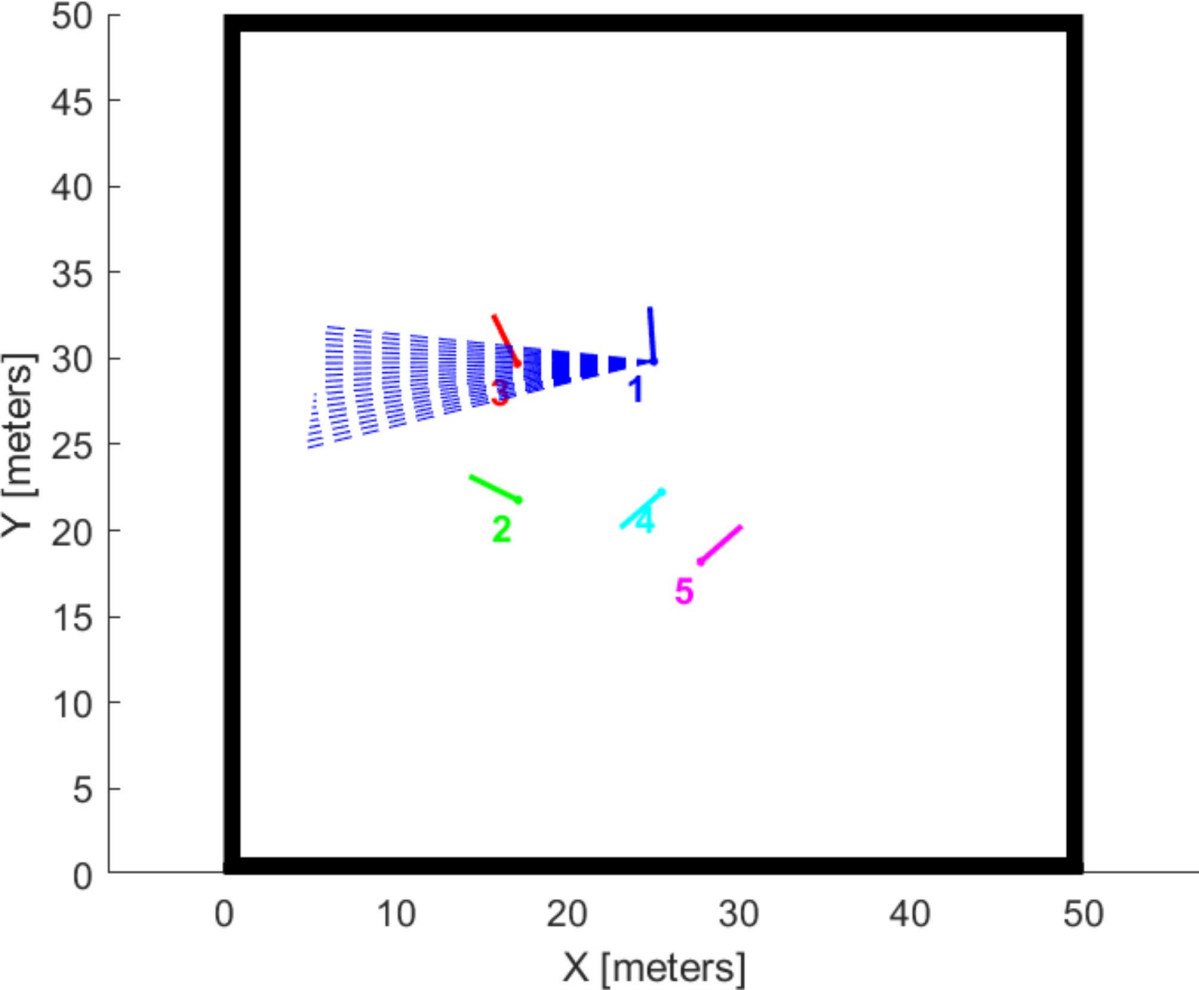
Robot 1 Lidar sensor rotation fault.

Mounting issues emerge from changes in the sensor’s position, which can be caused by improper fixation or vibrations during the robot’s movement. These issues can introduce drifting errors or offsets in LiDAR measurements. Additionally, defects in sensor subcomponents can arise from various causes. In this study, the simulated error is a malfunction in the LiDAR sensor rotation due to a failure in the sensor’s rotating motor or mechanism. This type of fault results in incomplete scans and limits the sensor’s coverage to a specific area as illustrated in Fig. 7, and consequently will affect the accuracy of mapping and detecting surrounding features.

### Formation reconfiguration

In the context of robotic formations, the process of formation reconfiguration occurs when the number of available robots within the formation changes. Each robot maintains knowledge of the current count of available robots through inter-robot communication. When a robot experiences a sensor fault, it activates a fault flag and communicates this information to other robots in the formation. Consequently, the remaining robots adjust their records regarding the currently available robots, leading to a modification in the geometrical shape of the formation. In this study, all robots are assigned a unique ID number from the set {1, 2, …, 5}. The robot with the smallest ID serves as the leader of the formation. If the leader (e.g., R_1_) encounters a fault and becomes isolated, the next robot with the smallest ID (e.g., R_2_) assumes the leader role. Additionally, all other available robots adapt their relative distances and orientations concerning the new leader (R_2_).

## Simulation and results

The control strategies previously discussed in Sects. 4 and 5 have been implemented in a multi-robot simulation environment using MATLAB/Simulink. The multi-robot system is comprised of five differential drive mobile robots. The simulation investigates diverse formation configuration scenarios, initially under fault-free conditions where the system adopts a pentagon-shaped formation. Subsequently, the second scenario replicates the first but introduces an obstacle. The third scenario explores the impact of a sensor fault on robot R5, resulting in the formation of a square shape. Lastly, the fourth scenario investigates a fault in the leader, leading the remaining robots to form a triangular shape.

### FDI dynamic threshold

**Fig. 8:**
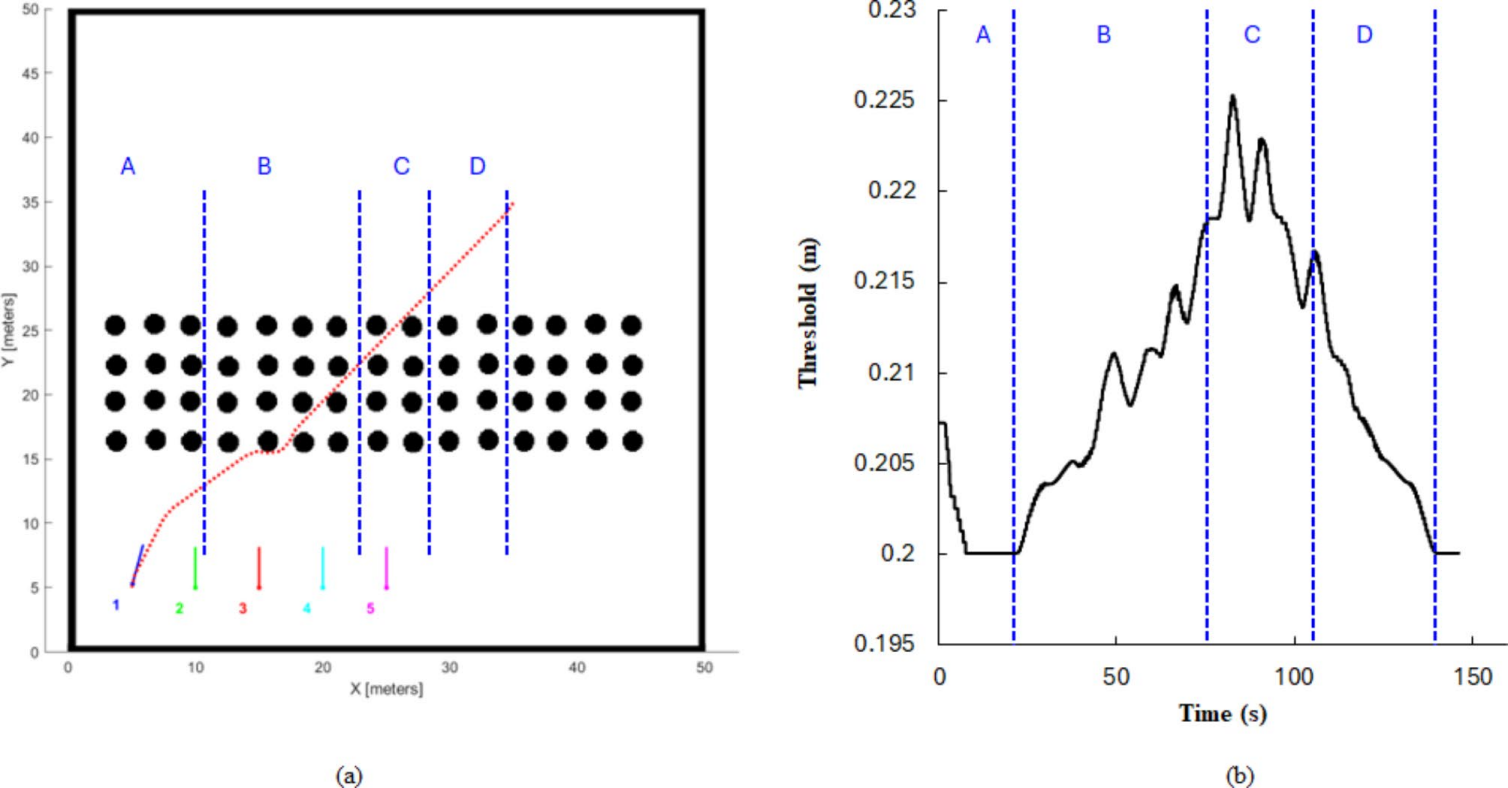
(**a**) Robot 1 trajectory through the region with obstacles, (**b**) 1 Robot 1 dynamic threshold from start to goal position.

In this section, the dynamic threshold proposed in Eq. (15) is simulated. The threshold value adjusts dynamically based on the degree of environmental clutter surrounding the robot. The cluttered region around the robot is quantified by computing the probability of occupied cells in the occupancy grid map. The baseline threshold is set to 0.2 m, a value determined by the accuracy limitations of the lidar sensor employed. For example, the Liadr “*rplidarA1”* has an accuracy of 2.5% of the actual distance (5–12 m)^47^. Robot (R1) initial position is set at coordinates (5, 5), with the goal position located at (35, 35). The robot’s trajectory traverses a region densely populated with obstacles, as depicted in Fig. 8 (a). The dynamic threshold algorithm adjusts the threshold value in accordance with the probability of occupied cells in the occupancy grid map. The threshold value increases proportionally to the concentration of obstacles in the robot’s proximal environment as illustrated in Fig. 8 (b).

The results demonstrate a dynamic adjustment of the threshold value in response to the robot’s proximity to obstacles and walls within the environment. As shown in sector A, as the robot traversed away from the map boundary, the threshold exhibited a decreasing trend. This decrease can be attributed to the diminishing influence of the wall as the robot distanced itself from that obstacle. Conversely, in sector B, an incremental rise in the threshold value was observed as the robot approached a region with a high density of obstacles. Furthermore, sector D witnessed a decline in the threshold value as the robot moved away from obstacles. These findings underscore the adaptability of the threshold parameter to the robot’s evolving surroundings, enabling dynamic adjustments to the FDI threshold based on the presence and proximity of obstacles within the environment.

### Fault-free, pentagon formation

In this scenario, the system is fault-free and robots start from a random position as shown in and tabulated in Considering R_1_ as the leader, the cost matrix employed in the Hungarian assignment algorithm to form the pentagon shape configuration from the initial positions is shown in (22), the columns represent the target positions relative to the leader R1, while the rows represent the remaining robots, excluding the leader.

22 $$\text{cost\,matrix}=\left[\begin{array}{cccc}10.18&15.79&21.47&21.12\\ 16.93&23.8&27.9&25.08\\ 21.94&27.22&25.9&19.16\\ 16.24&17.34&12.64&5.969\end{array}\right]$$

Since a decentralized control strategy is implemented, each robot minimizes its cost of travelling to the goal position by applying the Hungarian algorithm to the cost matrix. The x and y coordinates defining each robot’s goal position are calculated as shown in Table 1, the robot’s coordinates are based on the assigned distance and angle relative to the leader robot’s position. As previously illustrated in Fig. 3 (a), the linear Euclidean distances between the leader agent denoted as R_1_ and the follower agents R_2_ and R_5_ are 7.05 m, while the distances between the follower agents R_3_ and R_4_ are 11.41 m. Subsequently, each robot computes its trajectory to reach this goal pose by implementing a hybrid A* path planning algorithm.

**Table 1:** Robots’ starting position and goal position to form Pentagon configuration.

Robot ID	Starting positions			Goal position		Fault status
	x	y	ψ	x	y	
R_1_	25	25	π/2	25	25	False
R_2_	10	25	π/2	21.47	14.14	False
R_3_	10	35	π/2	19.29	20.85	False
R_4_	30	40	π/2	30.70	20.85	False
R_5_	35	25	π/2	28.52	14.14	False

**Fig. 9:**
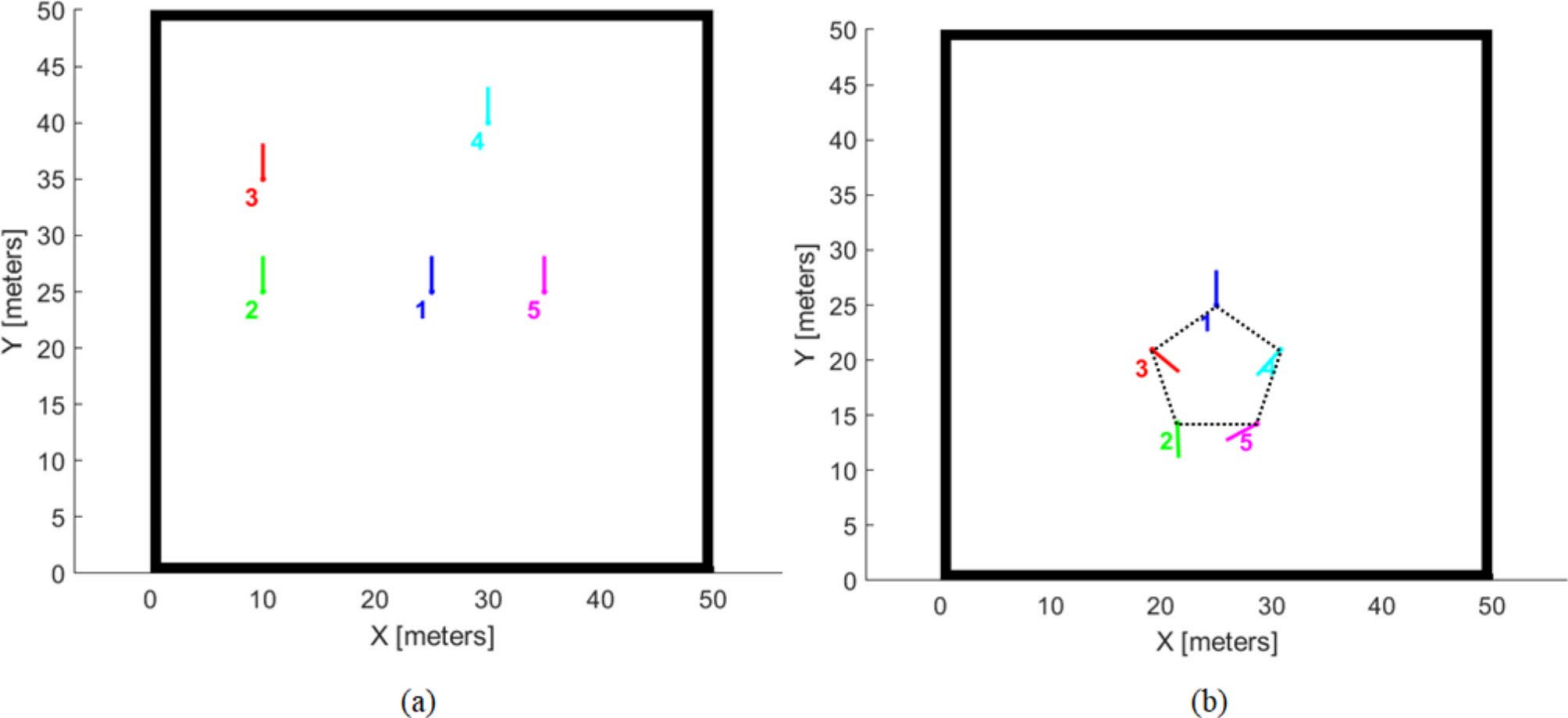
(**a**) Robot random initial pose, (**b**) robot final position in pentagon shape.

**Fig. 10:**
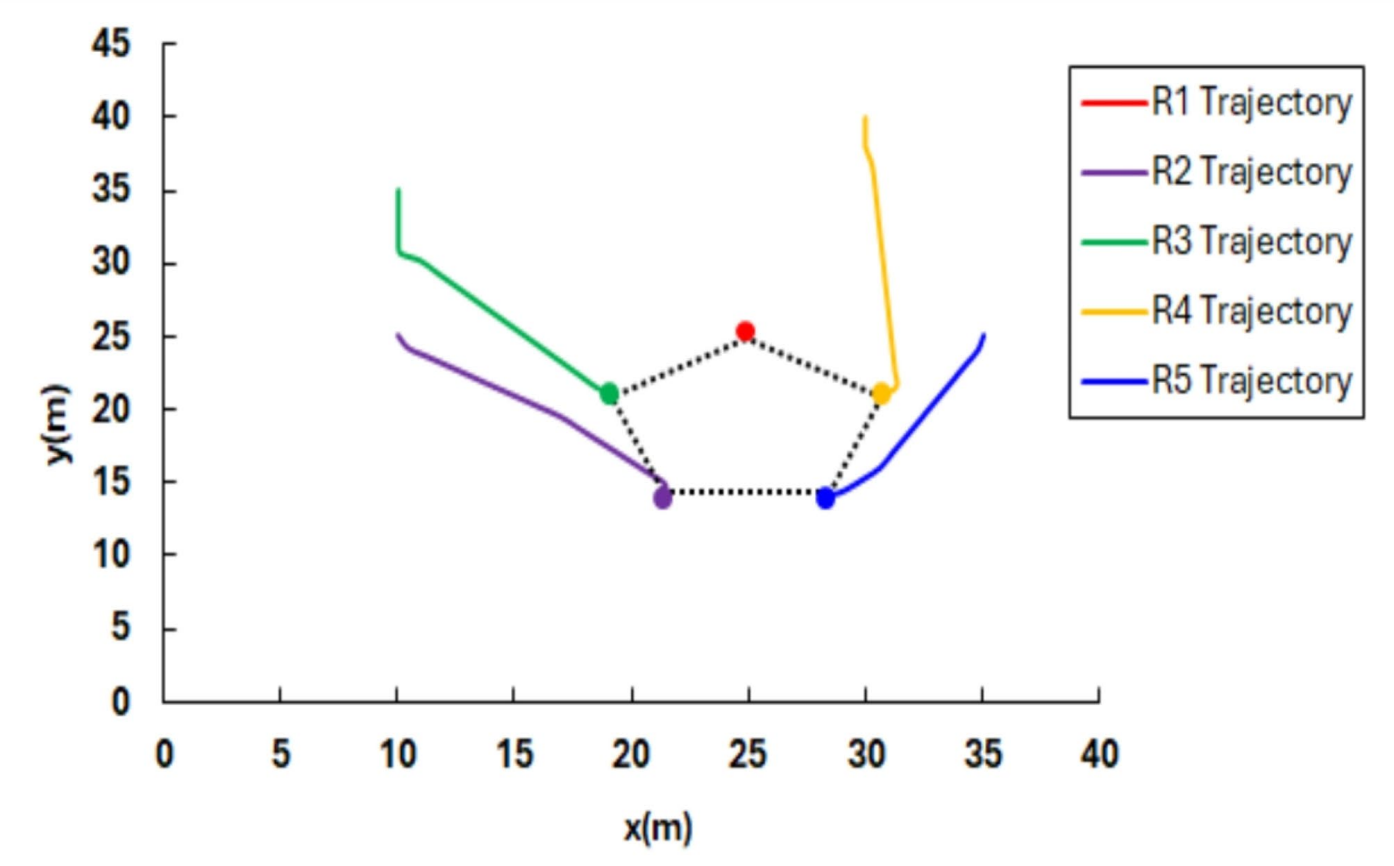
Robot’s trajectories to form pentagon shape configuration from start pose.

**Fig. 11:**
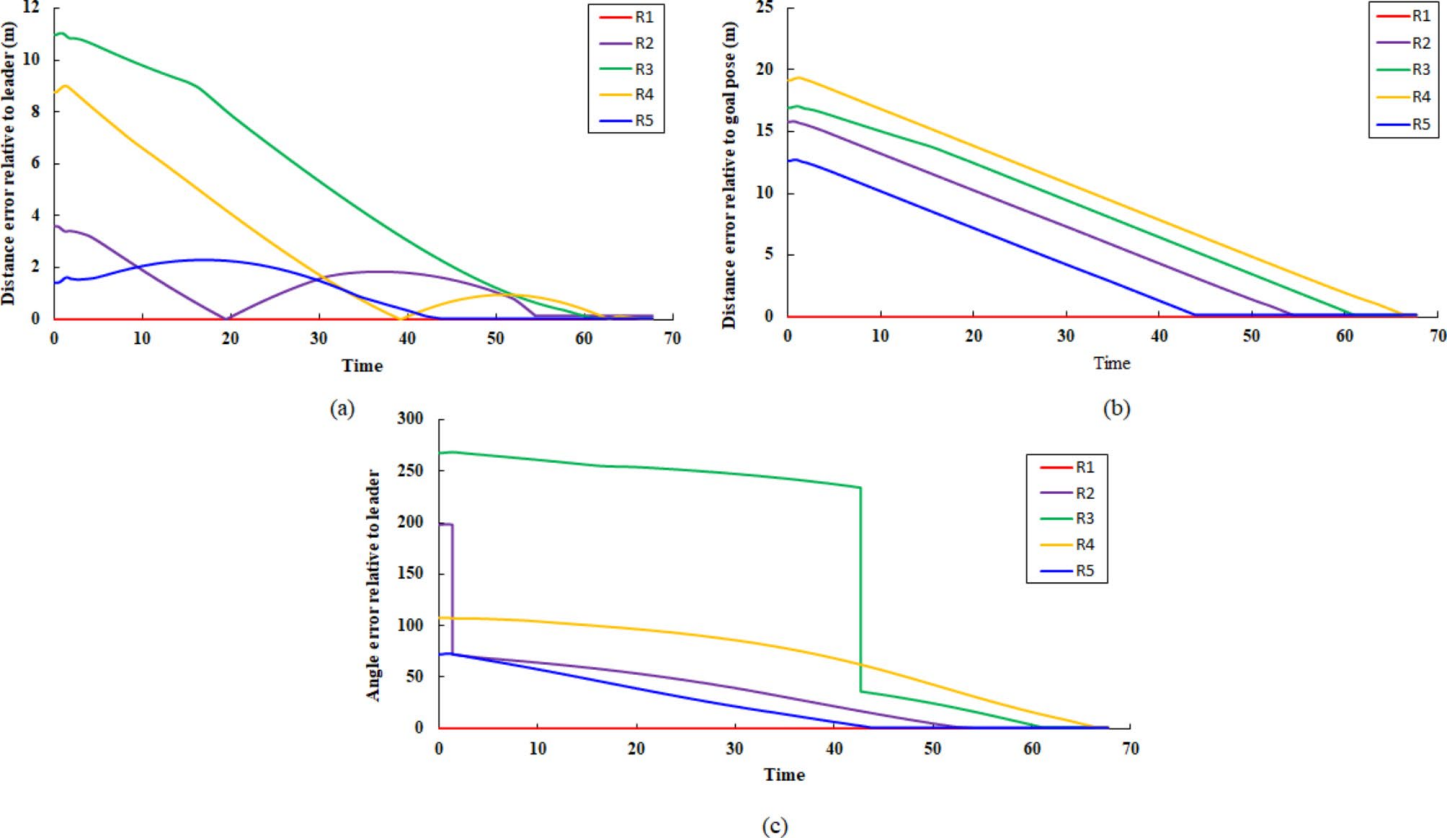
Pentagon shape formation error (**a**) distance error relative to the leader (m), (**b**) distance error to goal pose (m), and (**c**) orientation error relative to the leader (deg).

Figure 9(a) shows the initial positions of the robots, whereas Fig. 9 (b) shows the final goal positions attained by the agents, forming a pentagon-shaped formation. Furthermore, the computed trajectories executed by each agent to reach its respective goal position are illustrated in Fig. 10. In addition, Fig. 11 illustrates the error in three aspects; (a) Distance error relative to the leader robot, (b) Distance error relative to the goal position and (c) Orientation error relative to the leader. From the presented data it is evident that at t = 67.7 all the robotic entities converge to the assigned formation shape configuration.

**Fig. 12:**
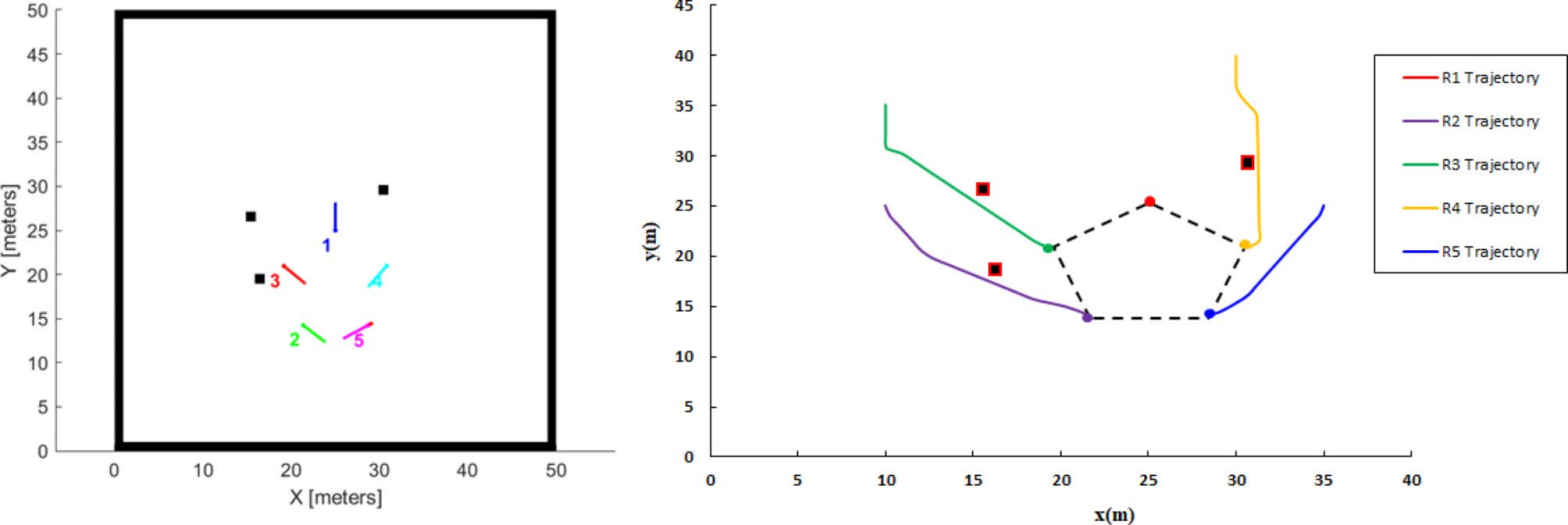
(**a**) Pentagon shape formation with obstacles in the surrounding environment, (**b**) Robot trajectory with encountering an obstacle in the environment Fault-free with an obstacle, pentagon formation.

In this particular scenario, the multi-robot system is evaluated for its ability to form a pentagonal formation configuration in the presence of obstacles along the formation path. Three obstacles are strategically positioned on the map at the following (x, y) coordinates: obs1 (15.5, 16.5), obs2 (30.5, 29.5), and obs3 (16.5, 19.5), as depicted in Fig. 12. The Vector Field Histogram (VFH) algorithm is employed for obstacle avoidance path planning, leveraging the range data obtained from the LiDAR sensor to detect obstacles in the surrounding environment. The results demonstrate that each robot follows a unique trajectory different from the initial scenario. A conspicuous distinction can be observed between the formation path planning depicted in Fig. 10, which represents an obstacle-free environment, and Fig. 12(b), where obstacles are present in the operational space. In addition, all robots successfully converge to the desired formation configuration.

### Robot 5 sensor fault and square formation

**Fig. 13:**
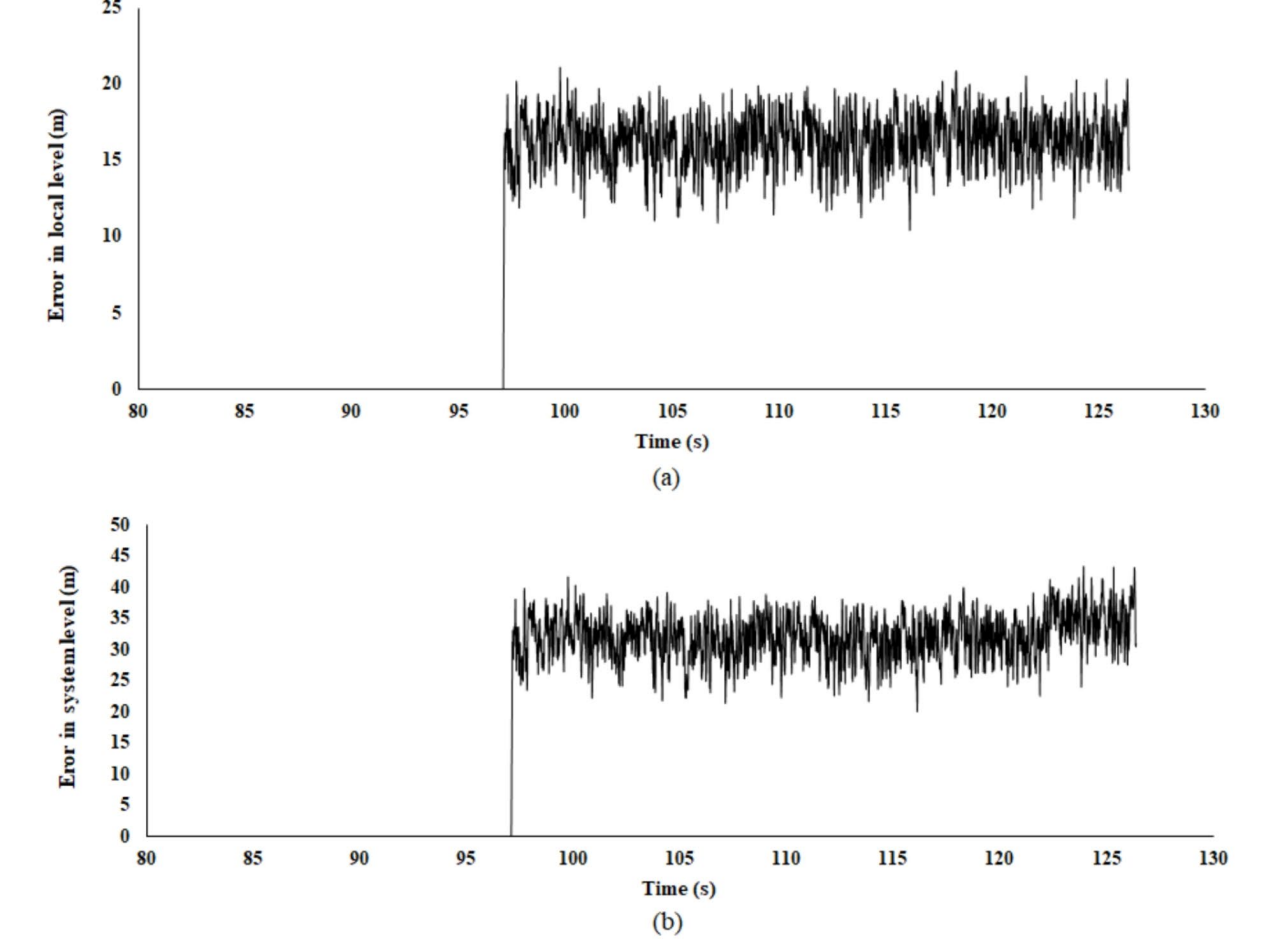
Error in (**a**) local level and (**b**) system level robot R5.

**Fig. 14:**
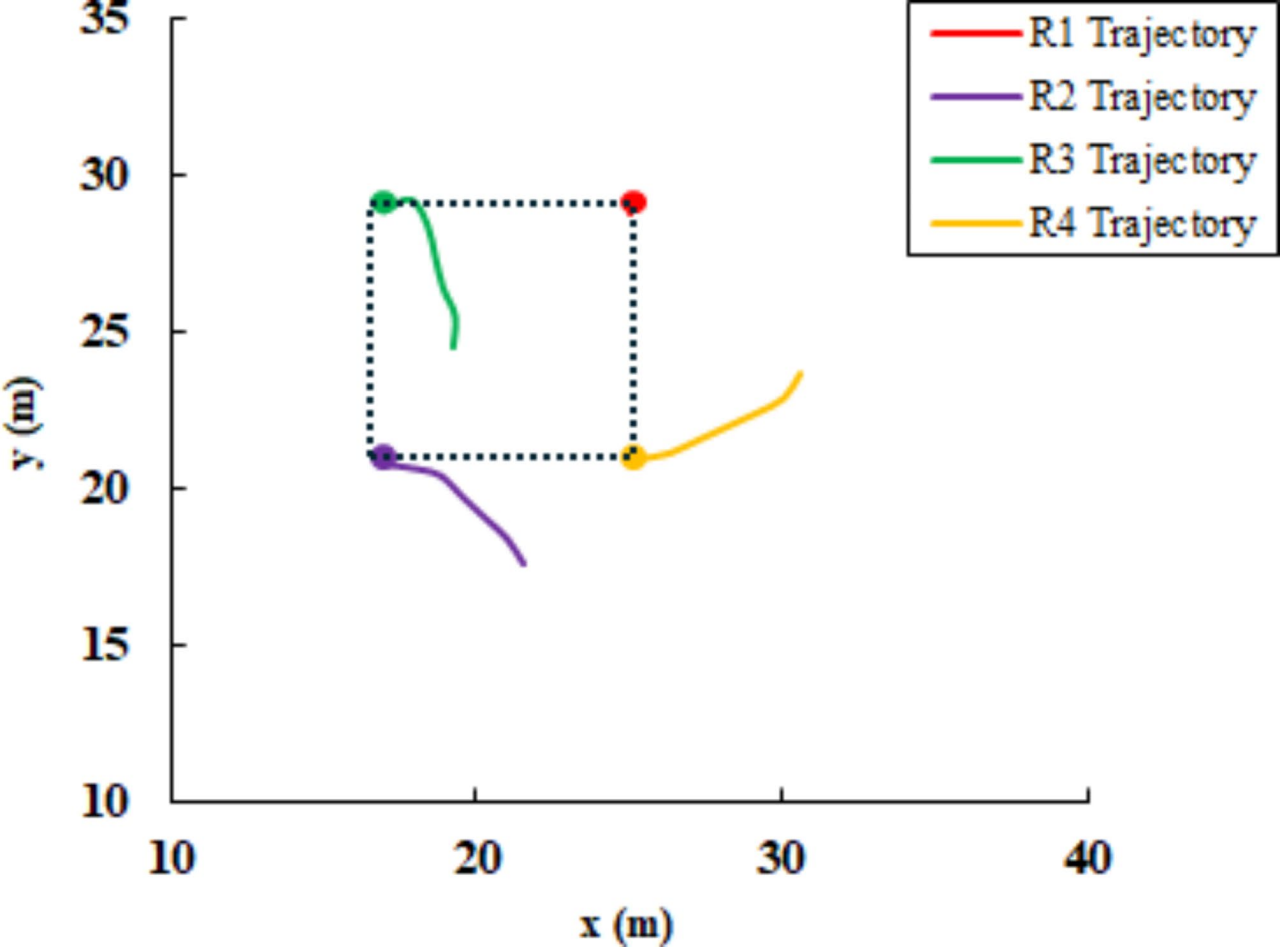
Robots trajectories for formation square shape formation.

The occurrence of LiDAR sensor faults can be attributed to various factors, as previously discussed. In the simulation model, these faults are simulated by introducing noise that emulates one of these potential causes. This section focuses on modelling the impact of smoke on the R_5_ LiDAR readings by manipulating the visibility conditions surrounding the robot. The effect of smoke is quantified using the visibility Eq. (18), which allows for a systematic analysis of sensor performance under degraded atmospheric conditions. At t = 92.5, robot R_5_ experienced a sensor fault, as illustrated in Fig. 13. The introduced noise resulted in an error on both the system and local levels. In addition, according to the introduced control strategy, robot (R_5_) triggers a fault flag to other robots in the multi-robot system to inform them that it experiencing a sensor fault and isolated from the formation. Then the robots in the system (R_1_, R_2_, R_3_ and R_4_) will update the information regarding the number of fault-free robots. Subsequently, with only four available robots, and with R_1_ assuming the role of the leader, each available robot performs task reassignment to allocate the robots for the establishment of a square formation configuration (Fig. 14). The corresponding cost matrix is shown in (23).

23 $$costmatrix=\left[\begin{array}{ccc}11.97&5.415&4.747\\ 4.612&4.464&6.951\\ 14.44&13.89&6.33\end{array}\right]$$

**Fig. 15:**
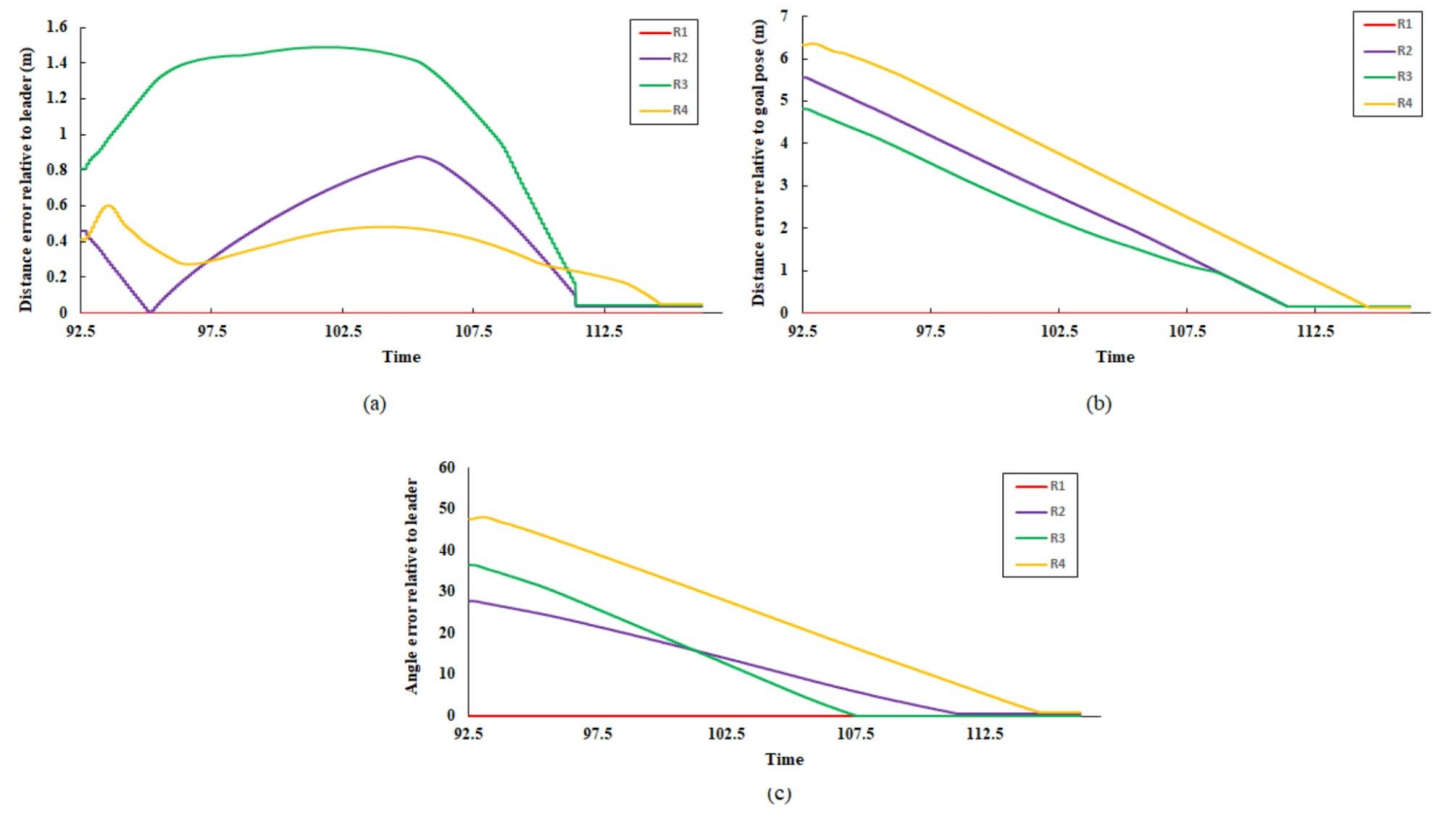
Square shape formation error (**a**) distance error relative to the leader (m), (**b**) distance error to goal pose (m), and (**c**) orientation error relative to the leader (deg).

At time step t = 115, the remaining robots successfully achieved convergence to the predetermined square formation configuration, as illustrated in Fig. 15. Robot R_5_ is isolated from the formation due to the aforementioned lidar sensor fault. The linear and angular velocity of R_5_ is set to zero. The complete trajectories of fault-free robots are presented in Fig. 14. The leader R_1_ pose is unchanged, while the other robot adjusts its position concerning R_1_. The robot’s final position in square shape formation and fault status are presented in Table 2.

**Table 2:** Robots position status in square shape formation.

Robot ID	Goal position		Fault status
	x	y	
R_1_	25	29.84	False
R_2_	17.21	20.79	False
R_3_	17.21	28.92	False
R_4_	25.1	20.85	False
R_5_	28.39	17.88	True

### Leader fault and triangle formation

In this scenario, at t = 117, a sensor subcomponent failure is deliberately introduced to R_1_. This failure includes a malfunction in lidar sensor rotation and it is applied by limiting the sensor range of scan as shown before in Fig. 7. This resulted in the classification of R_1_ as a faulty robot, alongside R_5_. Consequently, robots R_2_, R_3_, and R_4_ remained fault-free. As dictated by the implemented control strategy, due to the presence of only three available robots, the multi-robot system converged to a triangle-shaped formation configuration. Also, R_2_ assigned the leader role instead of R_1_. The cost matrix for assigning the target position to R_3_ and R_4_ is as follows:

24 $$\text{cost\,matrix}=\left[\begin{array}{cc}12.21&12.32\\ 11.42&6.136\end{array}\right]$$

**Fig. 16:**
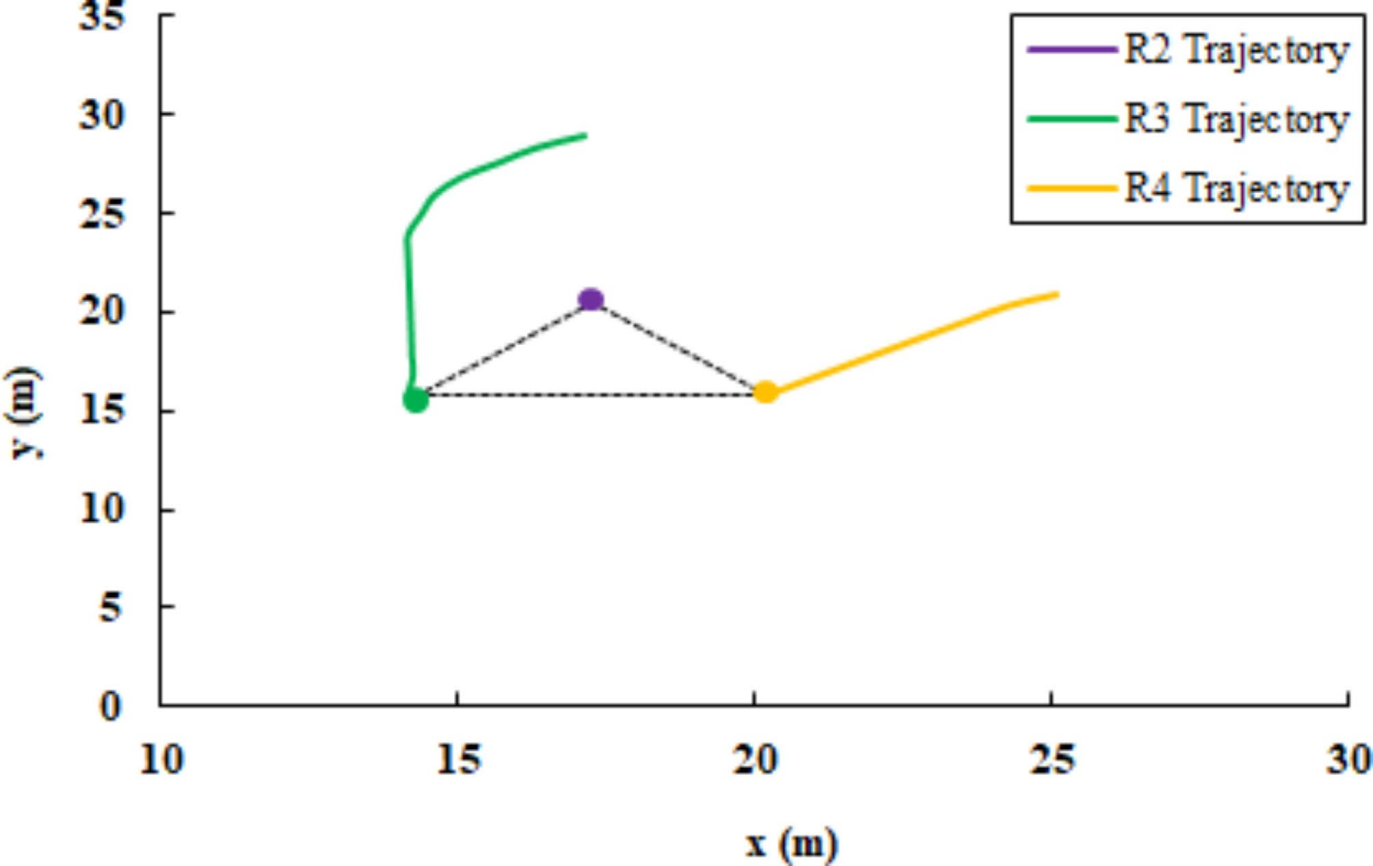
R3 and R4 trajectory to form triangle shape formation with R2 as leader.

**Fig. 17:**
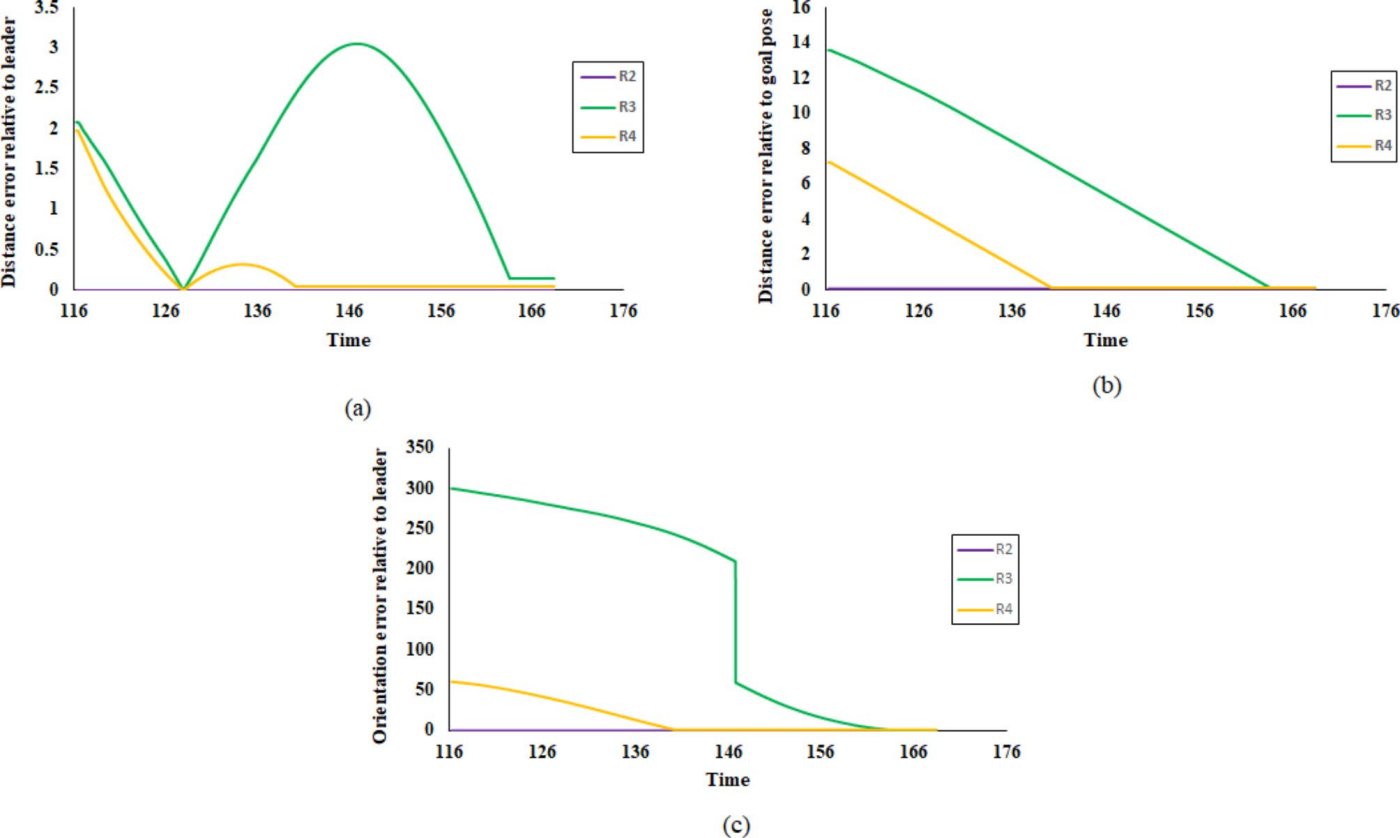
Triangle shape formation error (**a**) distance error relative to the leader (m), (**b**) distance error to goal pose (m), and (**c**) orientation error relative to the leader (deg).

The faulty robots (R_1_ and R_5_) are isolated from the formation and assigned a linear and angular velocity equal to zero. Additionally, Table 3. presents the final position of the agents after achieving their assigned positions. The poses of the faulty agents R_1_ and R_5_ have not changed since their last assigned positions. However, the available robots (R_2_, R_3_, and R_4_) form a triangular shape formation. Figure 16 depicts the trajectories of R3 and R_4_ to reach their goal positions relative to the leader robot R2. From the distance and orientation errors illustrated in Fig. 17, it can be observed that the available robots successfully converge to the assigned triangular shape formation and attain their goal positions.

**Table 3:** Robots position status in triangle shape formation.

Robot ID	Goal position		Fault status
	x	y	
R_1_	25	29.84	True
R_2_	17.21	20.79	False
R_3_	14.16	15.78	False
R_4_	20.25	15.75	False
R_5_	28.39	17.88	True

### Irregular polygon shape formation

**Fig. 18:**
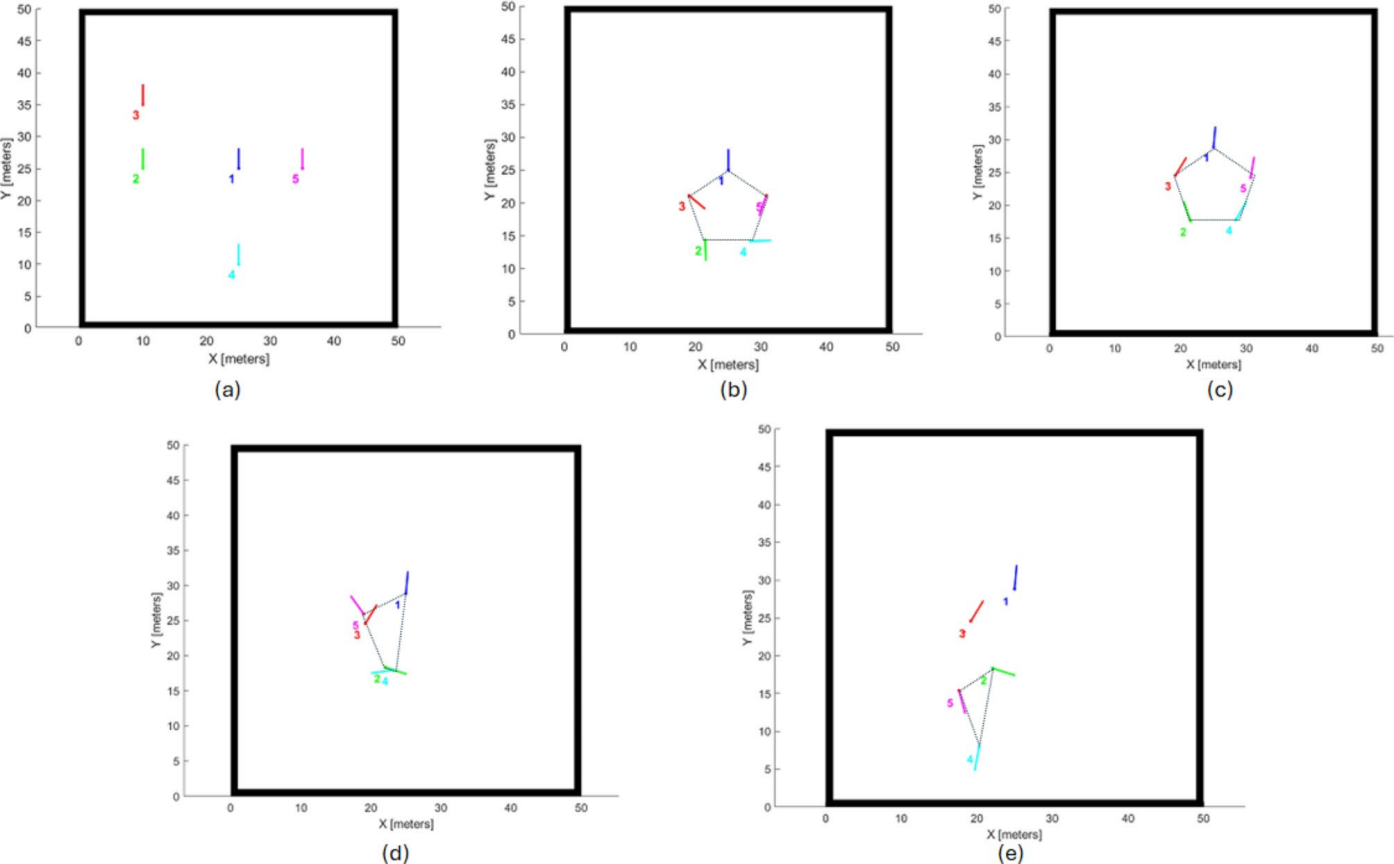
(**a**) Robots’ Initial positions, (**b**) Forming regular pentagon shape, (**c**) Moving the robot formation, (**d**) R3 fault and forming irregular quadrilateral shape formation, and (**e**) R1 fault, assign R2 leader role and forming irregular triangle shape formation.

In this simulation scenario, the robot initially starts from a random position as shown in Fig. 18 (a) and subsequently forms a regular pentagon shape as shown in Fig. 18 (b) and (c). At T = 86.9, Robot R_3_ experiences a sensor fault, causing it to become isolated from the formation. Consequently, the remaining robots, R_1_, R_2_, R_4_, and R_5_, form an irregular quadrilateral shape as show in Fig. 18 (d). Furthermore, at T = 133.2, the leader robot, R_1_, is subjected to a sensor fault and becomes isolated from the formation. As a result, R_2_ is assigned the leader role, and the available robots, R_2_, R_4_, and R_5_, form an irregular triangular shape as shown in Fig. 18 (e).

**Fig. 19:**
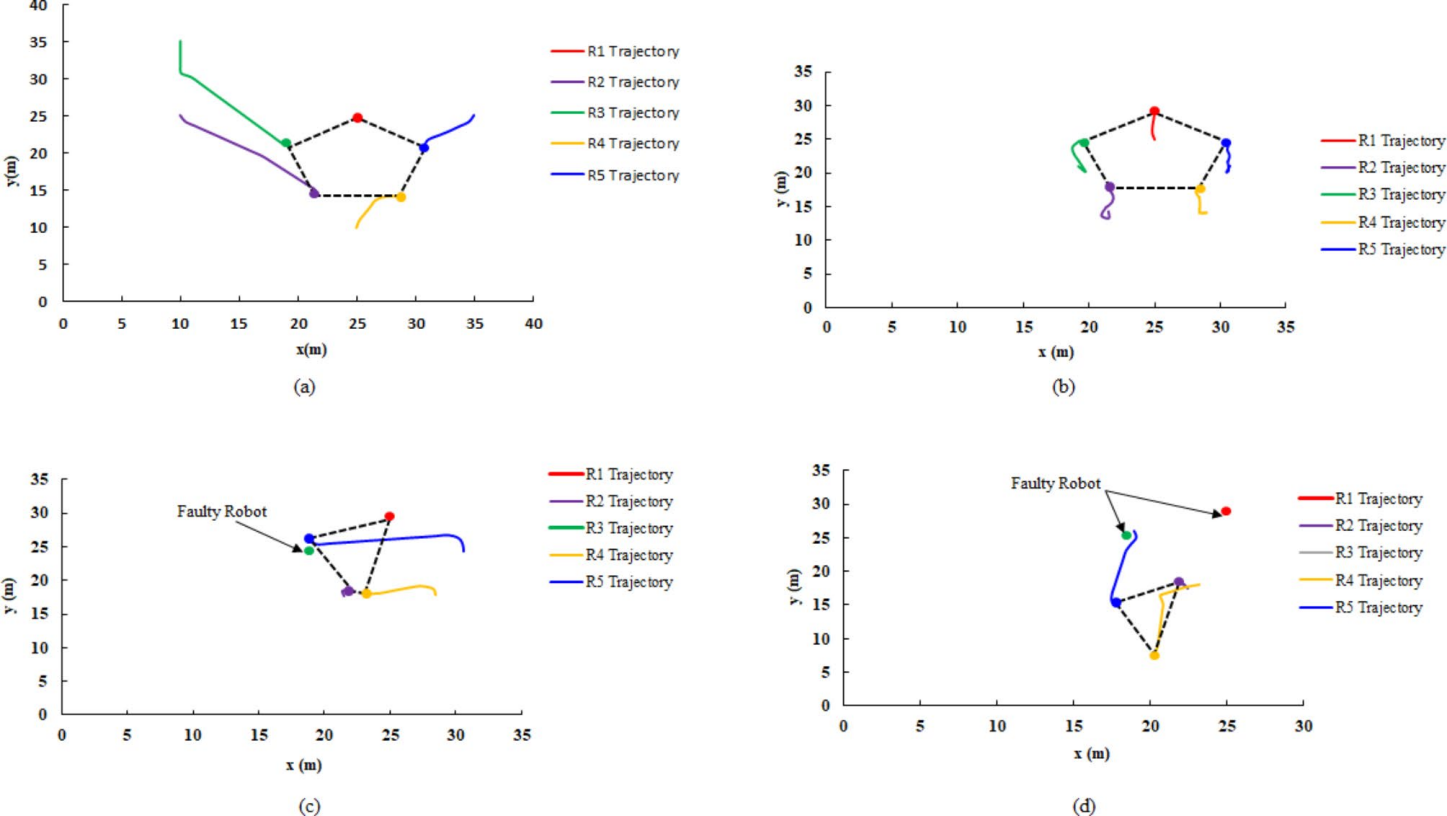
(**a**) Robots’ trajectory in forming pentagon shape formation, (**b**) Robots’ trajectory in moving in pentagon shape formation, (**c**) Robots’ trajectory in forming irregular quadrilateral shape formation and (**d**) Robots’ trajectory in forming irregular triangular shape formation.

**Fig. 20:**
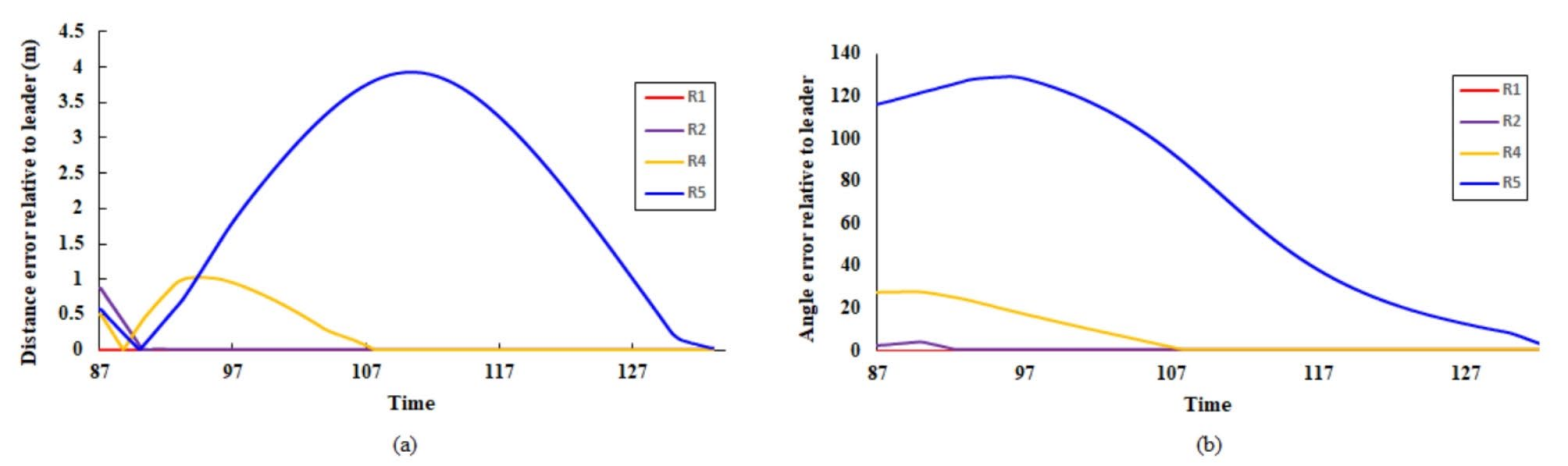
Distance and orientation error relative to the leader robot in forming irregular quadrilateral shape formation.

**Fig. 21:**
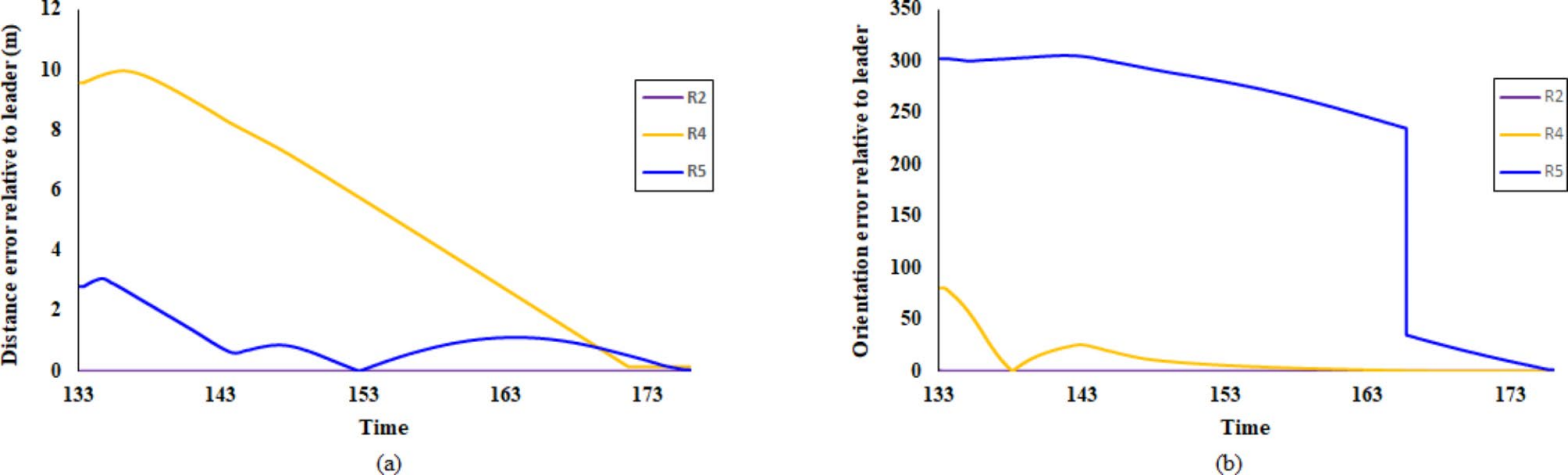
Distance and orientation error relative to the leader robot n forming irregular triangular shape formation.

The trajectories of the robots in the four scenarios—forming a pentagon shape, moving the pentagon shape formation, forming an irregular quadrilateral shape, and forming an irregular triangular shape—are illustrated in Fig. 19 (a), (b), (c), and (d), respectively. Additionally, Figs. 20 and 21 depict the distance and orientation errors relative to the leader robot in both irregular quadrilateral and triangular shape formation respectively. The robots successfully converge to the assigned shape formations.

## Conclusion

This paper presents the design and simulation of a Decentralized Fault-Tolerant Control (DFTC) scheme that accounts for lidar sensor faults. The proposed DFTC strategy is simulated on a multi-mobile robot system composed of a five-differential wheeled mobile robot platform. Each robot in the system is equipped with a wheel encoder, an Inertial Measurement Unit (IMU), and a lidar sensor. Fault Detection and Isolation (FDI) for the lidar sensor is achieved through two levels. The local level involves comparison with fused data from the wheel encoder and IMU sensor, and the system level involves comparison with data from lidar sensors mounted on other robots within the multi-robot system. The Fault Detection and Isolation (FDI) algorithm utilizes a dynamic threshold, which is determined by the percentage of clutter present in the surrounding environment. Furthermore, a moving average is employed in the computation of the residual at both the local and system levels of FDI. This proposed FDI technique is designed to ensure the detection of faults in lidar sensors and to effectively isolate any malfunctioning robots from the formation. Three types of lidar sensor faults were introduced including environmental conditions (smoke), subcomponent failure and mounting issues. Formation shape configurations are executed according to the number of available robots within the multi-robot system. Various simulation scenarios are implemented, and the results demonstrate that the robots successfully converge to the assigned formation shape configuration taking into consideration free-fault and faulty robots.

Prior research has addressed aspects of safety-critical control and fault tolerance in robotic systems^38^. presented a safety-critical control framework for a LiDAR-based system on a single robot platform. Furthermore^37^, investigated distributed fault-tolerant control (FTC) for multi-robot systems. However, the latter study did not specifically address LiDAR faults or malfunctions in multi robot system in its fault tolerance scheme.

In addition, the proposed Distributed Fault-Tolerant Control can be utilized in industrial settings, specifically in systems involving multiple robots for transportation purposes. This highlights the versatility and applicability of the DFTC in real-world scenarios. Owing to the constraints in funding, future work will focus on the practical implementation of the presented work in real-world scenarios. This includes the introduction of a novel control strategy designed to maintain the formation shape of multiple robots, even in the event of a fault occurrence leading to the isolation of a robot from its assigned formation. This approach ensures the robustness and resilience of the system in the face of unforeseen challenges.

## Data Availability

The data generated or analyzed during this study are included in this published article and for requesting any data contact the corresponding author Ahmed M. Elsayed (a.m.elsayed139@gmail.com).
